# Age-related secretion of grancalcin by macrophages induces skeletal stem/progenitor cell senescence during fracture healing

**DOI:** 10.1038/s41413-023-00309-1

**Published:** 2024-01-25

**Authors:** Nan-Yu Zou, Ran Liu, Mei Huang, Yu-Rui Jiao, Jie Wei, Yangzi Jiang, Wen-Zhen He, Min Huang, Yi-Li Xu, Ling Liu, Yu-Chen Sun, Mi Yang, Qi Guo, Yan Huang, Tian Su, Ye Xiao, Wei-Shan Wang, Chao Zeng, Guang-Hua Lei, Xiang-Hang Luo, Chang-Jun Li

**Affiliations:** 1 Department of Endocrinology, Endocrinology Research Center, Xiangya Hospital of Central South University, Changsha, Hunan 410008 China; 2 Department of Orthopaedics, Xiangya Hospital of Central South University, Changsha, Hunan 410008 China; 3 Department of Epidemiology and Health Statistics, Xiangya School of Public Health, Central South University, Changsha, China; 4 Hunan Key Laboratory of Joint Degeneration and Injury, Changsha, Hunan 410008 China; 5 Key Laboratory of Aging-related Bone and Joint Diseases Prevention and Treatment, Ministry of Education, Xiangya Hospital, Central South University, Changsha, China; 6 School of Biomedical Sciences, Institute for Tissue Engineering and Regenerative Medicine, Faculty of Medicine, The Chinese University of Hong Kong, Hong Kong SAR, China; 7 Department of Orthopaedics, The First Affiliated Hospital of Shihezi University, Shihezi, Xinjiang China; 8 National Clinical Research Center for Geriatric Disorders, Xiangya Hospital, Central South University, Changsha, Hunan 410008 China

**Keywords:** Metabolic bone disease, Bone quality and biomechanics

## Abstract

Skeletal stem/progenitor cell (SSPC) senescence is a major cause of decreased bone regenerative potential with aging, but the causes of SSPC senescence remain unclear. In this study, we revealed that macrophages in calluses secrete prosenescent factors, including grancalcin (GCA), during aging, which triggers SSPC senescence and impairs fracture healing. Local injection of human rGCA in young mice induced SSPC senescence and delayed fracture repair. Genetic deletion of *Gca* in monocytes/macrophages was sufficient to rejuvenate fracture repair in aged mice and alleviate SSPC senescence. Mechanistically, GCA binds to the plexin-B2 receptor and activates Arg2-mediated mitochondrial dysfunction, resulting in cellular senescence. Depletion of *Plxnb2* in SSPCs impaired fracture healing. Administration of GCA-neutralizing antibody enhanced fracture healing in aged mice. Thus, our study revealed that senescent macrophages within calluses secrete GCA to trigger SSPC secondary senescence, and GCA neutralization represents a promising therapy for nonunion or delayed union in elderly individuals.

## Introduction

Osteoporosis is characterized by low bone mass and destroyed microarchitecture, resulting in an increased risk of fracture.^1^ Epidemiological studies show that in the year 2000, there were estimated to be nearly 9 million osteoporotic fractures each year on a global scale, suggesting that almost one in two women and one in five men will experience a fracture in their lifetime from the age of 50 years.^2^ Additionally, decreased bone regenerative potential and a high risk of malunion, delayed union and nonunion in elderly individuals contribute to long-term disability or even death.^3,4^ However, the etiology of age-related impaired skeletal regenerative capacity remains incompletely understood.

Aged-related skeletal deterioration and impaired fracture healing are related to the accumulation of senescent cells, which are characterized by permanent cell cycle arrest, apoptosis resistance and a senescence-associated secretory phenotype (SASP).^5–8^ Targeting cellular senescence using pharmacological and genetic approaches prevents age-related bone loss in mice.^9^ Farr et al. revealed that myeloid cells within bone marrow and osteocytes in bone matrix become senescent and secrete SASP factors during aging.^10^ It has also been demonstrated that bone marrow adipocyte lineage cells undergo rapid cellular senescence after glucocorticoid treatment and secrete SASP factors to distribute cellular senescence of vascular endothelial cells and osteoprogenitors in the metaphysis of growing bone, leading to decreased peak bone mass, architectural deterioration, and increased fracture risk.^11,12^ Antagonizing the senescence of these cells improves glucocorticoid-induced bone deterioration.^11^ Our previous study found that senescent immune cells accumulated in the bone marrow and secreted grancalcin (GCA), which suppressed bone turnover and promoted marrow fat accumulation.^13^ Thus, accumulated senescent cells in the bone microenvironment play a significant role in skeletal aging, and eliminating senescent cells and/or blunting their SASP factors can prevent or delay age-related bone loss. Recent studies also suggested that the accumulation of senescent cells impaired fracture healing, and removing senescent cells by genetic and pharmacological approaches in aged mice improved fracture repair.^14–16^ However, the effects and underlying mechanisms of senescent cells in fracture healing during aging remain elusive.

Skeletal stem/progenitor cells (SSPCs) serve as osteochondro-precursors in the callus during fracture healing.^17^ Josephson et al. reported that the number of SSPCs decreases with age, leading to poor bone regeneration in elderly individuals in a population cohort.^18^ Studies have shown that SSPCs exert senescent phenotypes in aged mice, accompanied by decreased proliferation ability and diminished osteochondrogenic activity.^18–20^ The function of SSPCs within the callus is at least partially affected by local microenvironmental cues. Liu et al. reported that senescent cells in fracture calluses secrete SASP factors to inhibit the growth and proliferation of callus-derived mesenchymal progenitor cells and ultimately impair bone regeneration.^14^ However, the identification of a prosenescent niche, including primary senescent cells and prosenescent factors, within the callus that is responsible for SSPC senescence and dysfunction is far from clear.

Chronic inflammation is thought to be a major cause of the impaired regenerative capacity of the skeleton during aging, in contrast to well-balanced inflammation, which is needed for successful bone repair.^21^ The interaction between macrophages and SSPCs is critical for bone regeneration.^22^ Macrophages phagocytose necrotic cells and tissue debris at the fracture site and initiate the recruitment of periosteal mesenchymal stem cells, bone marrow mesenchymal stem cells, and vascular progenitor cells.^23,24^ Activated macrophages secrete chemokines to control the migration of SSPCs to calluses.^25,26^ Aging negatively affects the inflammatory response during fracture healing.^21^ Parabiosis and bone marrow transplantation studies have shown that macrophages from young mice secrete factors that rejuvenate fracture repair in aged mice.^27,28^ In contrast, macrophages from aged mice delay fracture healing in young mice.^29^ However, the factors secreted by aged macrophages remain elusive. Our previous studies have shown that senescent immune cells, including macrophages, release GCA to induce age-related bone loss.^13^ However, the effects of GCA secreted by infiltrating macrophages in calluses on fracture healing during aging need to be further explored.

Here, we show that macrophages release abundant quantities of GCA during aging to induce SSPC senescence, leading to the dysfunction of SSPCs and delayed fracture healing. Genetic deletion of *Gca* in monocytes/macrophages is sufficient to rejuvenate fracture repair in aged mice. In addition, Arg2-mediated mitochondrial dysfunction contributes to SSPC senescence induced by the GCA-plexin-B2 axis. Depletion of *Plxnb2* in SSPCs leads to SSPC dysfunction and senescence, thus impairing fracture healing in young mice. Moreover, GCA neutralization markedly enhances fracture healing ability in aged mice. Thus, our study reveals that senescent macrophages within the callus secrete GCA to induce SSPC senescence and impair fracture healing during aging, and local injection of a GCA-neutralizing antibody represents a promising therapy for impaired fracture repair in elderly individuals.

## Results

### Senescent SSPCs accumulate in calluses and impair fracture healing in aged mice

**Fig. 1:**
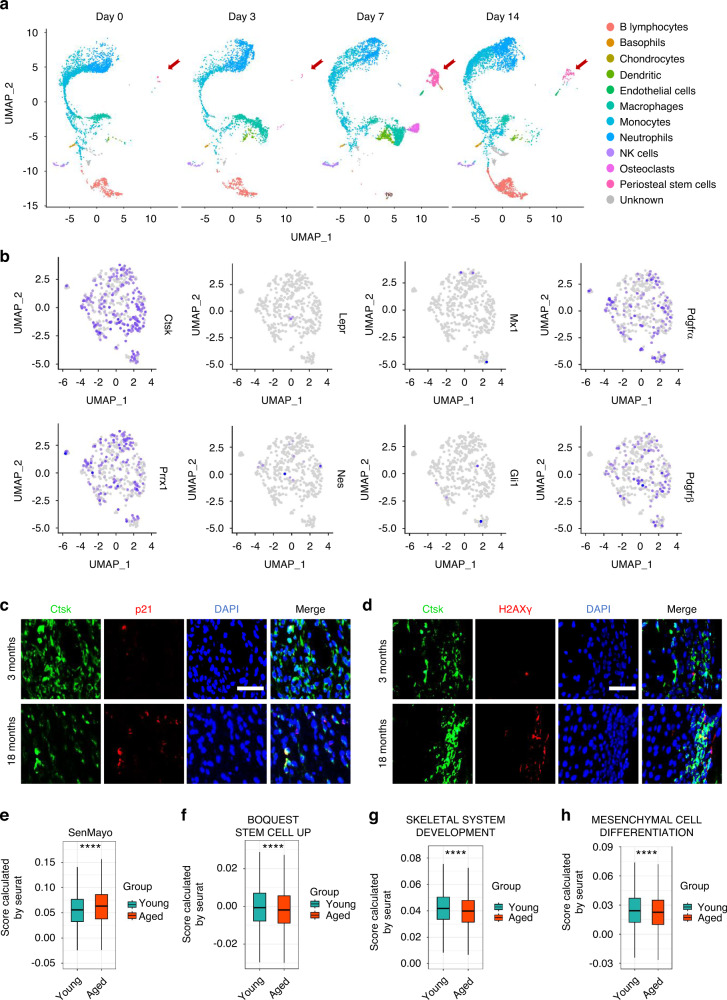
Senescent SSPCs accumulate in calluses and impair fracture healing in aged mice. **a**, **b** Uniform manifold approximation and projection (UMAP) plot showing 12 distinct clusters of cells identified (control group and on Days 3, 7, and 14). **b** Feature plots showing the expression distribution of marker genes of SSPC subsets on Day 7. Expression levels for each cell are color-coded and overlaid onto the UMAP plot. **c**, **d** Representative IF images in the periosteum of fracture callus at 10 days post-fracture, immunostained with Ctsk (green) and p21 (red) or γH2AX (red) antibodies and counterstained with DAPI (blue). Scale bars indicate 100 µm. **e**–**h** Boxplots demonstrating that aged SSPCs positively correlate with gene sets for senescence and negatively correlate with gene sets for stemness by the Wilcoxon test. Data are presented as the means ± SDs

To detect changes in fracture healing ability during aging, aged mice (18 months old) and young mice (3 months old) were subjected to transverse mid-diaphyseal femoral fractures, and healing was measured. Consistent with previous reports,^5^ aged mice showed obviously impaired fracture regeneration, as evidenced by smaller calluses and discontinuous cortical bone detected by microcomputed tomography (micro-CT) analysis, compared with young mice (Fig. S1a, b). Histological analysis indicated that aged mice displayed smaller areas of cartilage in the callus (Fig. S1c, d). Skeletal stem/progenitor cells (SSPCs), especially callus SSPCs, are indispensable for successful bone regeneration.^30–33^ The decreased number and function of SSPCs have been identified as potential causes of impaired tissue regeneration during aging.^18^ To identify the SSPC populations in calluses, we performed single-cell RNA sequencing (scRNA-seq) on callus tissues at different timepoints post-fracture.^34^ Periosteal stem cells were rarely detected before fracture (Day 0), increased at 3 days post-fracture (Day 3), and peaked at 7 days post-fracture (Day 7) (Fig. 1a and Fig. S1e, f). To label callus SSPCs in vivo, we compared the markers previously identified in labeled SSPCs,^31–33,35–39^ including *Ctsk, Prrx1, Pdgfrα, Pdgfrβ, Nes, LepR, Mx1* and *Gli1*, and found that the SSPCs with *Ctsk* expression accounted for the highest proportion of SSPCs in the fracture callus (Fig. 1b and Fig. S2a). Previous studies reported that *Ctsk* lineage cells in the periosteum were the major contributors to chondrocytes and osteoblasts during fracture healing.^31^ Thus, we selected Ctsk^+^ SSPCs for the subsequent study of fracture healing. To determine the senescence of SSPCs in calluses with age, we performed fluorescence staining in situ using antibodies against Ctsk and senescence markers (p21 and γH2AX) and found that the number of senescent Ctsk^+^ SSPCs was increased significantly in the calluses of aged mice compared with young mice (Fig. 1c, d and Fig. S2b, c). Previous studies found that the number of senescent cells increased in the fracture calluses of aged mice and caused inflammatory and senescent phenotypes.^18–20^ To explore the profile of senescent SSPCs in calluses during aging, we performed scRNA-seq on purified callus SSPCs from young (4-month-old) and aged (21-month-old) mice.^20^ Dominik et al. generated a senescence gene set (“SenMayo”) of 125 genes to identify senescent cells.^40^ SenMayo was validated to identify senescent cells at the single-cell level in murine bone/bone marrow and was confirmed to outperform existing senescence gene sets. Thus, we chose SenMayo to evaluate these callus SSPCs from young (4-month-old) and aged (21-month-old) mice. We found that in aged mice, senescence-associated genes were more highly enriched, *i.e*., had higher enrichment scores in the osteogenic and proliferating progenitors compared to those of young mice (Fig. 1e). Additionally, these callus progenitors showed decreased gene-set enrichment associated with stemness and osteogenesis (Fig. 1f–h). These data suggest that impaired skeletal regenerative capacity is accompanied by the dysfunction and senescence of SSPCs during aging.

### Grancalcin secreted by senescent macrophages in calluses induces SSPC senescence and delays bone regeneration

**Fig. 2:**
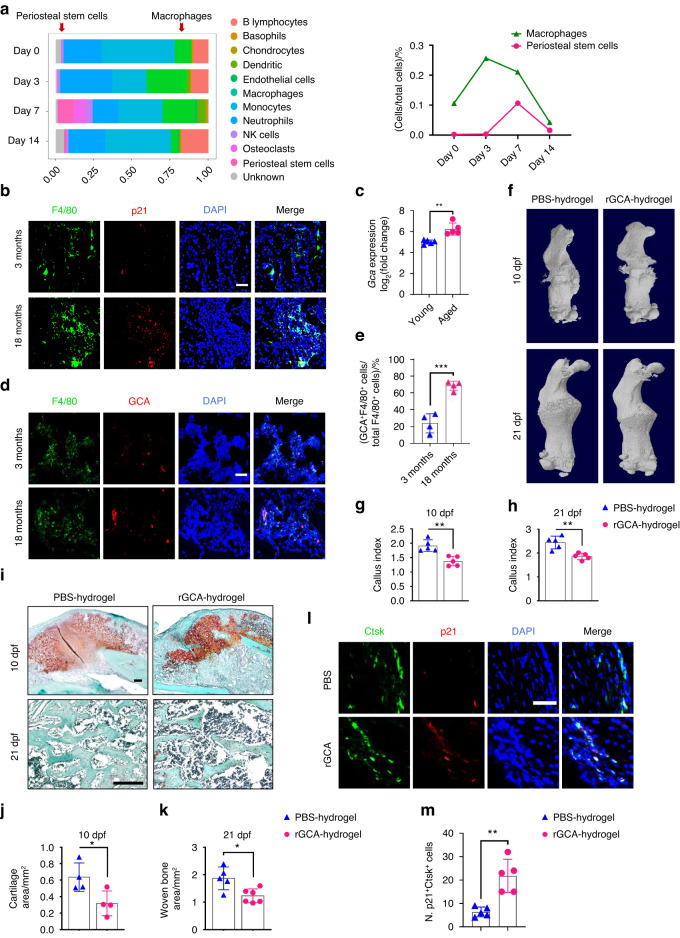
Aged macrophages in fracture calluses release GCA and impair bone regeneration. **a** Stacked bar chart showing the percentages of each cell type within callus tissue quantified on Day 0, Day 3, Day 7, and Day 14 post-fracture based on UMAP distribution. **b** Representative IF images of fracture calluses immunostained with F4/80 (green) and p21 (red) antibodies and counterstained with DAPI (blue) (*n* = 4). Scale bars indicate 100 µm. **c** Bioinformatics analysis of *Gca* gene expression levels in calluses from 5-month-old and 25-month-old mice at 5 days post-fracture (*n* = 5). **d** Representative IF images of fracture calluses immunostained with F4/80 (green) and GCA (red) antibodies and counterstained with DAPI (blue) (*n* = 4). Scale bars indicate 100 µm. **e** Quantification of GCA^+^F4/80^+^ cells (*n* = 4). **f** Representative micro-CT images of fractured femurs from PBS- and rGCA-treated mice at 10 dpf and 21 dpf. **g**, **h** The callus index of fractured femurs from PBS- and rGCA-treated mice at 10 dpf and 21 dpf (*n* = 5). **i**–**k** Safranin O staining showed cartilage callus formation and woven bone area from PBS- and rGCA-treated mouse fractured femurs at 10 dpf and 21 dpf (*n* = 4–6). Scale bar indicates 100 μm. **l** Representative IF images in the periosteum of the fracture callus at 10 days post-fracture, immunostained with Ctsk (green) and p21 (red) and counterstained with DAPI (blue). Scale bars indicate 100 µm. **m** Quantification of the number of p21- and Ctsk-positive cells per mm^2^ tissue area (N. p21^+^Ctsk^+^ cells) by Welch’s t test (*n* = 5). Data are presented as the means ± SDs. Unpaired t test, *P < 0.05 and **P < 0.01

**Fig. 3:**
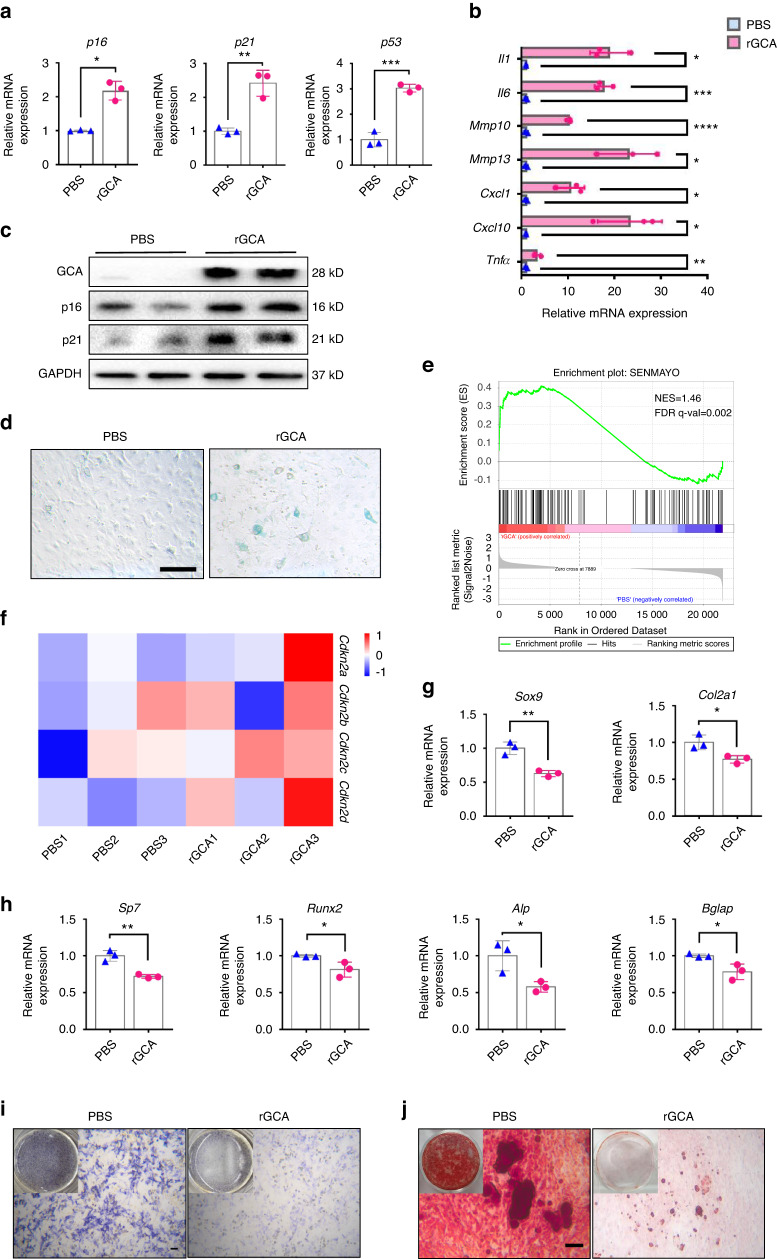
rGCA accelerates SSPC senescence and dysfunction. **a**, **b** RT‒PCR analysis of the gene expression of senescence markers and SASP factors in SSPCs (*n* = 3). *p16* by Welch’s t test. **c** Western blotting analysis of p16, p21 and GCA protein expression in PBS- or rGCA-treated SSPCs. **d** SA-β-Gal staining of SSPCs treated with PBS and rGCA. **e** GSEA plots demonstrating that SSPCs treated with rGCA positively correlate with gene sets for senescence (SenMayo). **f** Heatmap showing increased expression of INK family genes in rGCA-treated cells relative to PBS-treated cells. **g** RT‒PCR analysis of the gene expression of chondrogenesis markers in SSPCs (*n* = 3). **h** RT‒PCR analysis of the gene expression of osteogenesis markers in SSPCs (*n* = 3). **i**, **j** Representative images of ALP staining (**i**) and Alizarin Red staining (**j**) of SSPCs treated with PBS or rGCA. Scale bar indicates 100 μm. Data are presented as the means ± SDs. Unpaired t test. *P < 0.05, **P < 0.01 and ***P < 0.001

Next, we wondered which type of cells are the primary senescent cells that propagate senescence in SSPCs. The scRNA-seq data revealed that immune cells, particularly macrophages, responded quickly to fracture, and the population of macrophages peaked earlier than that of SSPCs in calluses (Fig. 2a). To explore whether macrophages accumulated in the callus become senescent with aging, we performed fluorescence staining in situ using antibodies against biomarkers for senescence-associated changes and F4/80^+^ macrophages in young and aged mice. We found that the percentage of p21^+^F4/80^+^ cells, as well as γH2AX^+^F4/80^+^ cells, increased in calluses with age (Fig. 2b and Fig. S3a–c). This observation prompted us to speculate that senescent macrophages at the fracture site secrete proinflammatory or prosenescence factors to create an inflammatory and senescent niche that leads to SSPC senescence. Previously, we reported that grancalcin (GCA) is mainly secreted by senescent macrophages and suppresses bone formation and resorption, leading to skeletal aging.^13^ In this study, we observed a higher level of GCA at the bone injury site in aged mice on 5 days post-fracture (5 dpf) than in young mice (Fig. 2c) (GEO: GSE99388). In addition, we compared GCA secreted by macrophages in the calluses of young and aged mice by performing fluorescence staining in situ using antibodies against GCA and F4/80. The number of GCA-expressing macrophages was higher in aged mice than in young controls (Fig. 2d, e). To determine whether GCA could induce SSPC senescence and lead to delayed fracture repair, we placed hydrogels containing human rGCA or PBS locally to transverse mid-diaphyseal femoral fractures of young mice. We detected a higher level of GCA at the bone injury site in mice administered rGCA than in PBS-treated controls (Fig. S3d, e). By micro-CT analysis, we observed a significantly reduced callus size in mice with administration of rGCA in comparison with PBS-treated controls at 10 dpf and 21 dpf (Fig. 2f–h). Smaller islands of cartilage and reduced woven bone area at the fracture site were detected in the calluses of rGCA-treated mice versus controls by Safranin O/Fast green staining analysis (Fig. 2i–k). Next, we measured the effects of rGCA on SSPC senescence. Immunofluorescence staining showed an increased number of senescent SSPCs in the calluses of rGCA-treated mice compared with PBS-treated mice, as characterized by increased p21- and γ-H2AX-positive SSPCs (Fig. 2l, m and Fig. S3f, g). To further test the direct effects of GCA on SSPC senescence, we harvested primary mouse SSPCs and treated these cells with rGCA. qPCR analysis revealed that SSPCs treated with rGCA showed higher expression of genes associated with senescence and SASP factors (Fig. 3a, b). Western blotting analysis and SA-β-gal staining also demonstrated that rGCA treatment led to SSPC senescence (Fig. 3c, d). We then performed RNA sequencing on SSPCs treated with PBS/rGCA. Consistent with previous results, the key senescence genes *Cdkn2a*, *Cdkn2c*, and *Cdkn2d* and the “SenMayo” gene set displayed highly significant enrichment in rGCA-treated SSPCs (Fig. 3e, f). In addition, we observed a diminished osteochondrogenic capacity of SSPCs with rGCA administration in contrast to the PBS-treated controls (Fig. 3g–j). Taken together, these data indicate that GCA secreted by senescent macrophages induces SSPC senescence, impairs the function of SSPCs and delays fracture healing.

### Genetic deletion of *Gca* in macrophages mitigates SSPC senescence and rejuvenates fracture repair in aged mice

**Fig. 4:**
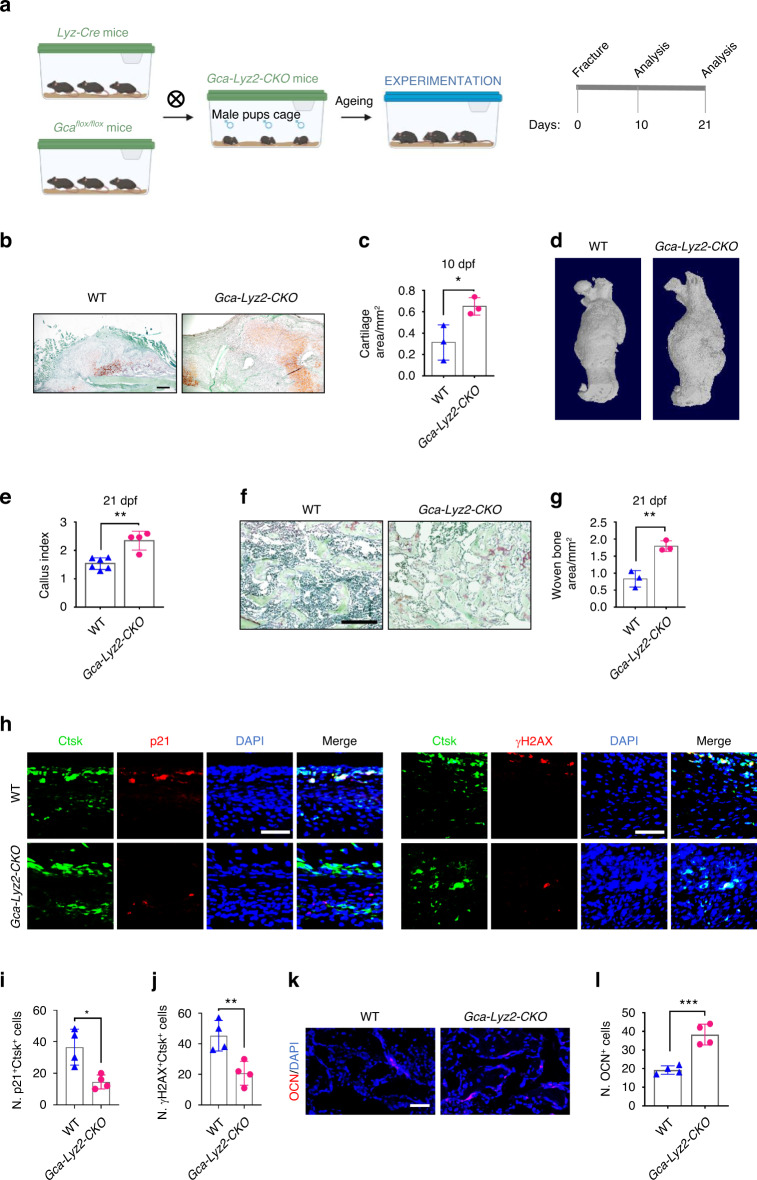
Genetic deletion of *Gca* in macrophages rejuvenates fracture repair in aged mice. **a** Schematic of experiments on *Gca-Lyz2-CKO* mice. **b**, **c** Safranin O staining showing cartilage callus formation in aged *Gca-Lyz2-CKO* mice and *Gca*^*flox/flox*^ control mice (WT) at 10 dpf (*n* = 3). Scale bar indicates 100 μm. **d** Representative micro-CT images of fractured femurs in *Gca-Lyz2-CKO* mice and WT mice at 21 dpf. **e** The callus index of fractured femurs from *Gca-Lyz2-CKO* mice and WT mice at 21 dpf (*n* = 4–6). **f**, **g** Safranin O staining showing the woven bone area of aged *Gca-Lyz2-CKO* mice and *Gca*^*flox/flox*^ control mice (WT) at 21 dpf (*n* = 3). Scale bar indicates 100 μm. **h** Representative IF images in the periosteum of the fracture callus at 10 days post-fracture, immunostained with Ctsk (green) and p21 (red) or γH2AX (red) antibodies and counterstained with DAPI (blue). Scale bars indicate 100 µm. **i**, **j** Quantification of the number of p21- and Ctsk-positive cells per mm^2^ tissue area (N. p21^+^Ctsk^+^ cells) by Welch’s t test and the number of γH2AX- and Ctsk-positive cells per mm^2^ tissue area (N. γH2AX^+^Ctsk^+^ cells) (*n* = 4). **k** Representative IF images of fracture calluses at 21 days post-fracture, immunostained with Ocn (red) antibodies and counterstained with DAPI (blue). Scale bars indicate 100 µm. **l** Quantification of the number of Ocn-positive cells per mm^2^ tissue area (N. Ocn^+^ cells) (*n* = 4). Data are presented as the means ± SDs. Unpaired t test, *P < 0.05, **P < 0.01 and ***P < 0.001

The above data prompted us to test whether the deletion of *Gca* in macrophages rejuvenates fracture repair in aged mice. We crossed *Gca*^*flox/flox*^ mice with *Lyz2-Cre* mice to generate mice with deletion of the *Gca* gene in myeloid lineage cells (*Gca-Lyz2-CKO*) (Fig. 4a).^41,42^ The number of GCA-expressing macrophages in the calluses of *Gca-Lyz2-CKO* mice was decreased by 61.9% compared with that in *Gca*^*flox/flox*^ mice (Fig. S4a, b). We observed significantly increased cartilage formation in aged (18-month-old) *Gca-Lyz2-CKO* mice compared to age-matched *Gca*^*flox/flox*^ control mice (WT) (Fig. 4b, c). Subsequently, micro-CT reconstruction of fractured bones demonstrated a robust bony hard callus in aged *Gca-Lyz2-CKO* mice at 21 dpf (Fig. 4d, e). Safranin O/Fast green staining analysis revealed a larger callus size and more bone trabeculae in calluses from aged *Gca-Lyz2-CKO* mice than in those from control mice (Fig. 4f, g). To determine whether *Gca* knockout in fracture calluses would affect SSPC senescence, we performed immunofluorescence in situ in fracture calluses at 10 dpf. As expected, the number of senescent SSPCs decreased significantly in the calluses of *Gca-Lyz2-CKO* mice compared with *Gca* ^*flox/flox*^ mice (Fig. 4h–j). In addition, an increased number of osteocalcin (Ocn)-positive osteoblastic cells was observed in the calluses of *Gca-Lyz2-CKO* mice compared with those of *Gca* ^*flox/flox*^ mice (Fig. 4k, l). These results show that deletion of *Gca* in macrophages is able to mitigate SSPC senescence in calluses and promote fracture repair in aged mice.

### GCA binds to the plexin-B2 receptor to induce SSPC senescence

**Fig. 5:**
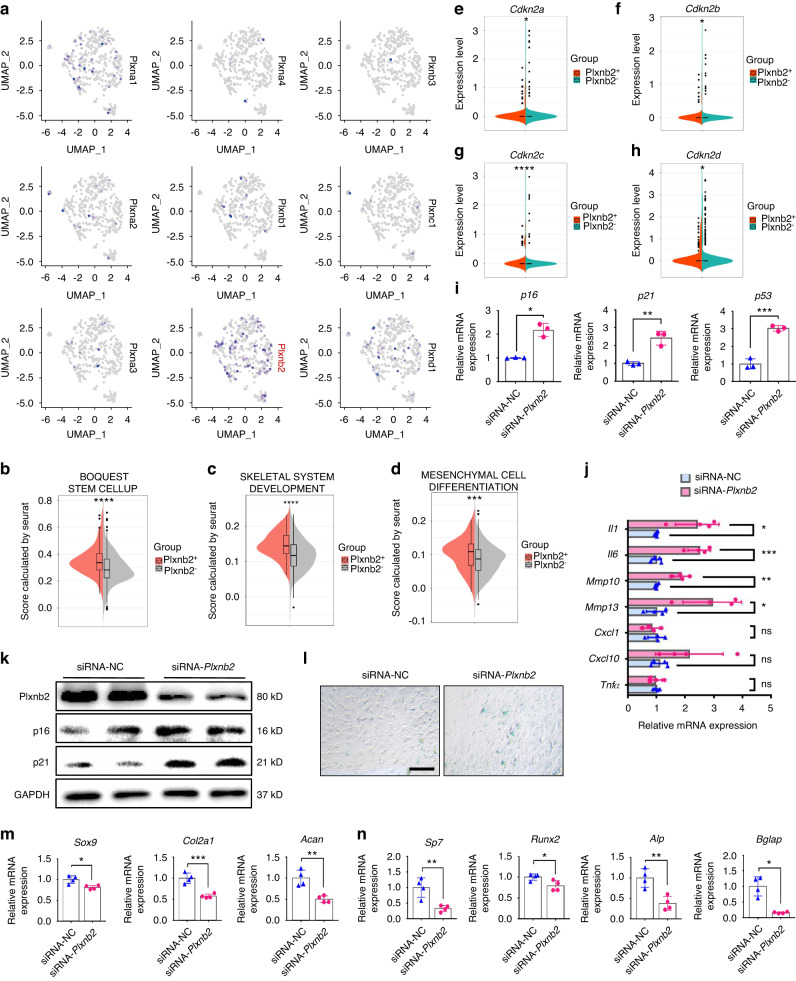
Knockout of *Plxnb2* accelerates SSPC senescence and dysfunction. **a** Feature plots showing the expression distribution of the plexin family on Day 7. Expression levels for each cell are color-coded and overlaid onto the UMAP plot. **b**–**d** Violin plots demonstrating that *Plxnb2*^+^ SSPCs positively correlate with gene sets for stemness. **e**–**h** Violin plots demonstrating that *Plxnb2*^-^ SSPCs positively correlate with INK family genes. **i**, **j** QPCR analysis of senescence marker and SASP factor gene expression in SSPCs (*n* = 3–4). *p16* and *Il1* by Welch’s t test. **k** Western blotting analysis of p16, p21 and PLXNB2 protein expression in siRNA-NC- or siRNA-*Plxnb2-*transfected SSPCs. **l** SA-β-Gal staining of siRNA-NC- or siRNA-*Plxnb2-*transfected SSPCs. Scale bar indicates 100 μm. **m** RT‒PCR analysis of chondrogenesis marker gene expression in SSPCs (*n* = 4). **n** RT‒PCR analysis of osteogenic marker gene expression in SSPCs (*n* = 4). *Bglap* by Welch’s t test. Data are presented as the means ± SDs. Unpaired t test. *P < 0.05, **P < 0.01, ***P < 0.001 and ns indicates no significant difference

Next, we further explored the downstream signaling that mediates the effects of GCA on SSPCs. Previously, we found that plexin-B2 (PLXNB2) is a functional receptor of GCA in BMSCs regulating osteogenesis.^13,43^ Additionally, it has been reported that PLXNB2 is expressed in vascular endothelial cells and protects bone blood vessels from senescence.^11^ Thus, we speculate that PLXNB2 may also mediate the effects of GCA on SSPC senescence. PLXNB2 belongs to the plexin family.^44^ We analyzed the distribution of plexin family members in callus SSPCs at different timepoints following fracture by conducting scRNA-seq.^34^ Notably, we found that the expression of *Plxnb2* was highest in SSPCs compared with other members of the plexin family (Fig. 5a and Fig. S5a). Then, we divided SSPCs into *Plxnb2*-positive SSPCs and *Plxnb2*-negative SSPCs (Fig. S5b). We found that *Plxnb2*-positive SSPCs showed higher expression of genes associated with stemness, stem cell differentiation and skeletal development than *Plxnb2*-negative SSPCs (Fig. 5b–d and Fig. S5c). In contrast, senescence-related genes, such as *Cdkn2a*, *Cdkn2b*, *Cdkn2c*, and *Cdkn2d*, were enriched in *Plxnb2*-negative callus SSPCs (Fig. 5e–h). To validate the direct effects of PLXNB2 on SSPCs, SSPCs were subjected to *Plxnb2* siRNA transfection, and the senescence phenotype of SSPCs was then measured. Higher expression of senescence-related genes and an increased number of SA-β-gal^+^ cells were detected in the SSPCs transfected with *Plxnb2* siRNA relative to control siRNA-treated SSPCs (Fig. 5i–l and Fig. S5d). We then examined whether knockdown of *Plxnb2* would lead to the diminished osteochondrogenic capacity of SSPCs. We found that SSPCs transfected with *Plxnb2* siRNA showed decreased expression of chondrogenic genes (*Sox9*, *Col2a1* and *Acan*) and osteogenic genes (*Sp7*, *Runx2*, *Alp* and *Bglap*) (Fig. 5m, n).

**Fig. 6:**
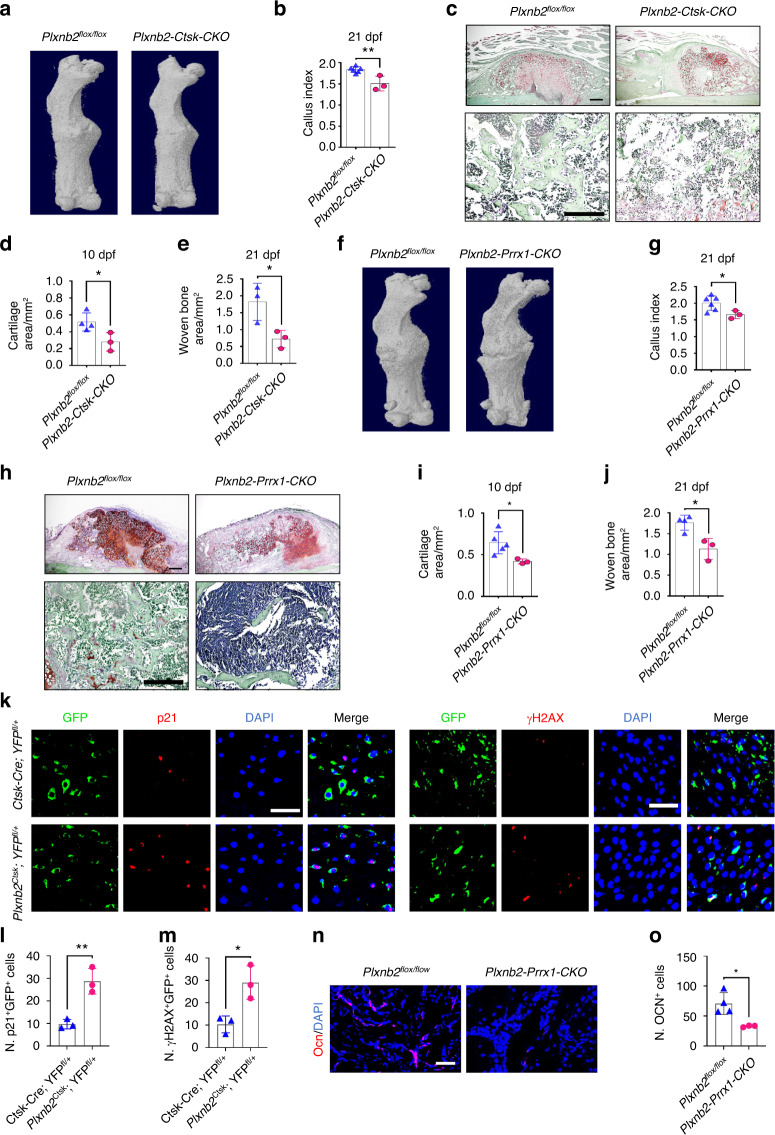
Deletion of *Plxnb2* in Ctsk^+^ and Prrx1^+^ cells impairs fracture repair in adult mice. **a** Representative micro-CT images of fractured femurs from *Plxnb2-Ctsk-CKO* mice and *Plxnb2*^*flox/flox*^ control mice at 21 dpf. **b** The callus index of fractured femurs from *Plxnb2-Ctsk-CKO* mice and *Plxnb2*^*flox/flox*^ mice at 21 dpf (*n* = 3–6). **c**–**e** Safranin O staining showing cartilage callus formation and woven bone area of fractured femurs from *Plxnb2-Ctsk-CKO* mice and *Plxnb2*^*flox/flox*^ mice at 10 dpf and 21 dpf (*n* = 3–4). Scale bar indicates 100 μm. **f** Representative micro-CT images of fractured femurs from *Plxnb2-Prrx1-CKO* mice and *Plxnb2*^*flox/flox*^ control mice at 21 dpf. **g** The callus index of fractured femurs from *Plxnb2-Prrx1-CKO* mice and *Plxnb2*^*flox/flox*^ mice at 21 dpf (*n* = 3–6). **h**–**j** Safranin O staining showing cartilage callus formation and woven bone area of fractured femurs from *Plxnb2-Prrx1-CKO* mice and *Plxnb2*^*flox/flox*^ mice at 10 dpf and 21 dpf (*n* = 3–5). Scale bar indicates 100 μm. **k** Representative IF images of fracture calluses at 10 days post-fracture, immunostained with GFP (green) and p21 (red) or H2AXγ (red) antibodies and counterstained with DAPI (blue). Scale bars indicate 100 µm. **l**, **m** Quantification of the number of p21- and GFP-positive cells per mm^2^ tissue area (N. p21^+^GFP^+^ cells) and the number of γH2AX- and GFP-positive cells per mm^2^ tissue area (N. γH2AX^+^GFP^+^ cells) (*n* = 3). **n** Representative IF images of fracture calluses at 21 days post-fracture, immunostained with Ocn (red) antibodies and counterstained with DAPI (blue). Scale bars indicate 100 µm. **o** Quantification of the number of Ocn-positive cells per mm^2^ tissue area by Welch’s t-test (N. Ocn^+^ cells) (*n* = 3–4). Data are presented as the means ± SDs. Unpaired t test, *P < 0.05 and **P < 0.01

To further investigate the effects of PLXNB2 in SSPCs on fracture repair during aging in vivo, we obtained mice with deletion of the *Plxnb2* gene in Ctsk^+^ SSPCs by crossing *Plxnb2*^*flox/flox*^ mice with *Ctsk-Cre* mice*. Plxnb2-Ctsk-CKO* mice and control mice were subjected to transverse mid-diaphyseal femoral fractures. Fracture repair was impaired in *Plxnb2-Ctsk-CKO* mice compared to control mice. Micro-CT analysis indicated a smaller and less mineralized callus in *Plxnb2-Ctsk-CKO* mice compared with control littermates (Fig. 6a, b and Fig. S6a). Additionally, we performed histological analysis in *Plxnb2-Ctsk-CKO* mice and control mice. We found that the area of cartilage and woven bone in calluses was significantly decreased in *Plxnb2-Ctsk-CKO* mice at 10 dpf and 21 dpf (Fig. 6c–e). To further exclude the effects of PLXNB2 in Ctsk^+^ non-SSPCs on fracture healing, we generated a mouse model with knockout of the *Plxnb2* gene in Prrx1^+^ SSPCs (*Plxnb2-Prrx1-CKO* mice) according to the high expression of *Prrx1* genes in SSPCs in calluses, as shown in Fig. 1b. Smaller calluses and smaller areas of cartilage and woven bone were also observed in *Plxnb2-Prrx1-CKO* mice than in control mice (Fig. 6f–j and Fig. S6b).

To trace the changes in *Ctsk* lineage cells at the fracture site in response to *Plxnb2* deficiency in vivo, we crossed *Plxnb2-Ctsk-CKO* mice with *YFP* reporter mice to generate *Ctsk-Cre*; *Plxnb2* ^*flox/flox*^; *YFP*^*flox/+*^ mice (*Plxnb2*^*Ctsk*^*; YFP*^*fl/+*^), and *Ctsk-Cre; YFP*^*fl/+*^ mice served as controls. Costaining of GFP with senescence markers (p21 and γ-H2AX) showed that the number of senescent GFP^+^ SSPCs increased significantly in the calluses of mice with *Plxnb2* knockdown in Ctsk^+^ lineage cells (Fig. 6k–m and Fig. S6c). In addition, a decreased number of Ocn-positive osteoblasts was observed in the calluses of *Plxnb2-Prrx1-CKO* mice compared with *Plxnb2*^*flox/flox*^ mice (Fig. 4n, o). Thus, these data suggested that depletion of *Plxnb2* leads to SSPC senescence and impaired fracture repair.

### The GCA-PLXNB2 axis may promote SSPC senescence through Arg2-mediated mitochondrial dysfunction

**Fig. 7:**
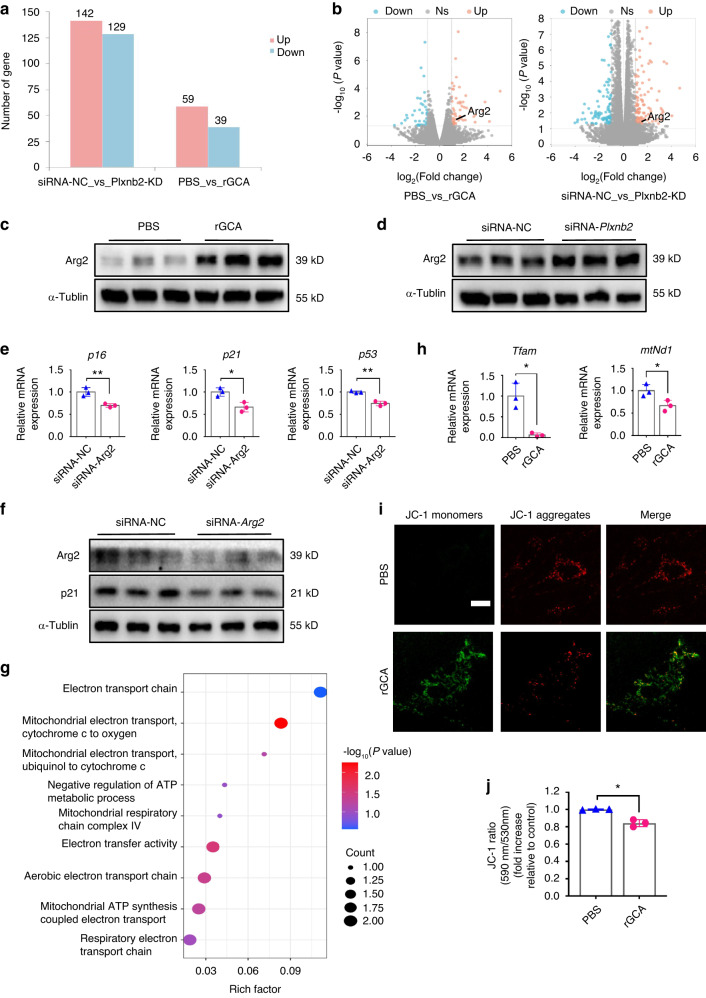
The GCA-PLXNB2 axis may promote SSPC senescence through the mitochondrial enzyme Arg2. **a** Statistics of differentially expressed genes in the rGCA and/or siRNA-*Plxnb2* groups ( ≥ 2-fold change). **b** Volcano plots showing increased *Arg2* expression in the rGCA and/or siRNA-*Plxnb2* groups ( > 2-fold change). **c**, **d** Western blotting analysis of Arg2 protein expression in the rGCA and/or siRNA-*Plxnb2* groups. **e** RT‒PCR analysis of senescence marker gene expression in siRNA-NC and siRNA-*Arg2* SSPCs (*n* = 3). **f** Western blotting analysis of Arg2 and p21 protein expression in the siRNA-NC and siRNA-*Arg2* groups. **g** GO analysis of differentially expressed genes related to mitochondrial function. **h** RT‒PCR analysis of *Tfam* and *mtNd1* gene expression in PBS- and rGCA-treated SSPCs (*n* = 3). *Tfam* by Welch’s t test. **i** Fluorescence microscopy image showing the JC-1 probe in PBS- and rGCA-treated SSPCs. Scale bars indicate 20 µm. **j** Mitochondrial membrane potential was measured using JC1 staining by Welch’s t test. Data are presented as the means ± SDs. Unpaired t test, *P < 0.05 and **P < 0.01

To identify the downstream pathway of PLXNB2 signaling regulated by GCA in SSPCs, we performed RNA sequencing on SSPCs treated with rGCA and SSPCs transfected with siRNA-*Plxnb2*. The analysis of data from rGCA-treated versus PBS-treated SSPCs identified 59 upregulated genes and 39 downregulated genes (Fig. 7a). Additionally, we found 142 genes with upregulated expression and 129 genes with downregulated expression in SSPCs transfected with siRNA-*Plxnb2* compared with controls (Fig. 7a). To identify shared downstream factors of rGCA-treated SSPCs and siRNA-*Plxnb2*-transfected SSPCs, we performed collective analysis of the combined data from rGCA- and PBS-treated SSPCs and siRNA-*Plxnb2*- and siRNA-control-transfected SSPCs. A total of 7 differentially expressed factors with at least a 2-fold change were identified, including *Oasl1*, *Colgalt2*, *Slco4a1*, *Arg2*, *Adora3*, *Nyx* and *Isg15* (Fig. S7a). Among them, arginase-II (Arg2) has been reported to promote endothelial cell senescence and vascular smooth muscle cell senescence.^45–47^ *Arg2* deficiency plays an important role in lifespan extension in mice.^48^ Thus, we chose Arg2 for further study. We verified significantly increased mRNA and protein levels of Arg2 in rGCA-treated and siRNA-*Plxnb2*-transfected SSPCs (Fig. 7b–d). To test whether Arg2 could influence SSPC senescence, we treated SSPCs with H_2_O_2_ to induce senescence and transfected SSPCs with siRNA*-Arg2*. We observed decreased senescence-related gene expression in SSPCs transfected with *Arg2-*siRNA compared to the control siRNA group (Fig. 7e, f and Fig. S7b-f). Arg2 is a mitochondrial enzyme and has been reported to be involved in mitochondrial dysfunction.^49–51^ To better dissect the role of mitochondrial dysfunction in GCA-induced SSPC senescence, we compared differentially expressed genes (DEGs) associated with mitochondrial function in SSPCs treated with rGCA or PBS. Gene Ontology (GO) analysis revealed that these DEGs were highly related to the “mitochondrial electron transport, cytochrome c to oxygen”, “mitochondrial ATP synthesis coupled electron transport”, and “mitochondrial respiratory chain complex IV” pathways, which were notable for their close links to mitochondrial function (Fig. 7g). We then examined key genes related to mitochondrial biogenesis and glycolysis in vitro and found sharply decreased expression in SSPCs treated with rGCA (Fig. 7h and Fig. S7g). In addition, rGCA treatment decreased the mitochondrial transmembrane potential (Fig. 7i, j). These data suggest that Arg2-mediated mitochondrial dysfunction may be an important mechanism of SSPC senescence regulated by the GCA-PLXNB2 axis.

### GCA-neutralizing antibody restores SSPC function and bone regeneration in aged mice

**Fig. 8:**
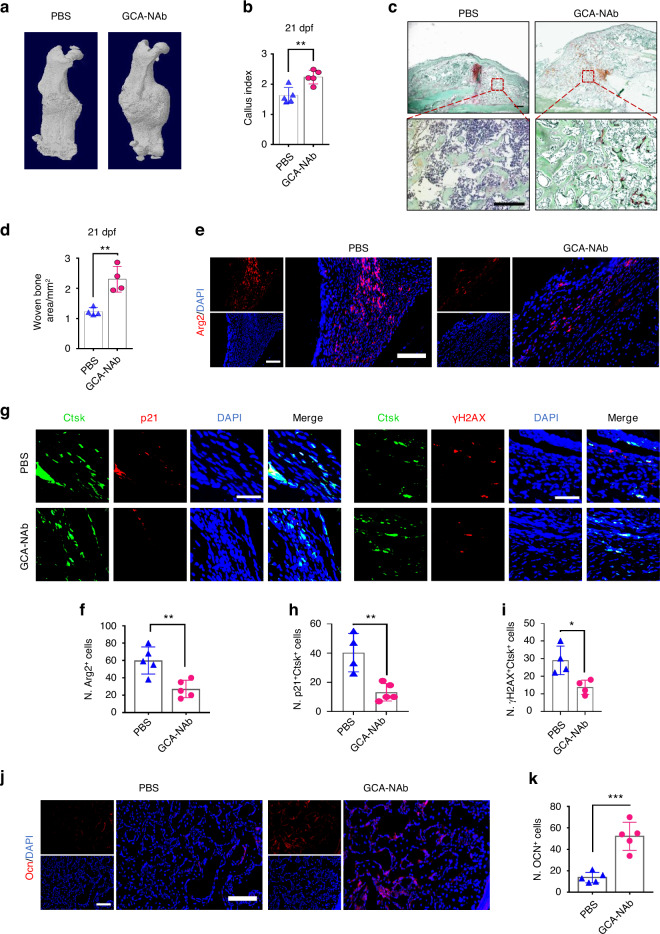
GCA-neutralizing antibody restores bone regeneration in aged mice. **a** Representative micro-CT images of fractured femurs from GCA-NAb- and PBS-treated mice at 21 dpf. **b** The callus index of fractured femurs from GCA-NAb- and PBS-treated mice at 21 dpf (*n* = 5). **c**, **d** Safranin O staining showing the woven bone area in calluses of fractured femurs from GCA-NAb- and PBS-treated mice at 21 dpf (*n* = 4). Scale bar indicates 100 μm. **e** Representative IF images of fracture calluses at 21 days post-fracture, immunostained with Arg2 (red) antibodies and counterstained with DAPI (blue). Scale bars indicate 100 µm. **f** Quantification of the number of Arg2-positive cells per mm^2^ tissue area (N. Arg2^+^ cells) (*n* = 5). **g** Representative IF images of the periosteum of fracture calluses immunostained with Ctsk (green) and p21 (red) or γH2AX (red) antibodies and counterstained with DAPI (blue). Scale bars indicate 100 µm. **h**, **i** Quantification of the number of p21- and Ctsk-positive cells per mm^2^ tissue area (N. p21^+^Ctsk^+^ cells) by Welch’s t test and the number of γH2AX- and Ctsk-positive cells per mm^2^ tissue area (N. γH2AX^+^Ctsk^+^ cells) (*n* = 4). **j** Representative IF images of fracture calluses at 21 days post-fracture, immunostained with Ocn (red) antibodies and counterstained with DAPI (blue). Scale bars indicate 100 µm. **k** Quantification of the number of Ocn-positive cells per mm^2^ tissue area (N. Ocn^+^ cells) (*n* = 5). Data are presented as the means ± SDs. Unpaired t test, *P < 0.05, **P < 0.01

The above data indicate that GCA secreted by senescent macrophages accelerates SSPC senescence and impairs fracture repair. We then tested whether a neutralizing antibody against GCA (GCA-NAb) could promote fracture healing in aged mice. Our previous studies screened a GCA-NAb and confirmed its therapeutic potential for treating bone loss in old mice.^13^ To further investigate the therapeutic effects of GCA-NAb in fracture repair during aging, we treated aged mice with GCA-NAb locally at the fracture sites twice a week for 21 days. Consistent with our findings that genetic ablation of *Gca* is beneficial to fracture repair, we found that treatment with GCA-NAb restored fracture repair in aged mice. Micro-CT analysis demonstrated that GCA-NAb improved the formation of mineralized calluses relative to vehicle-treated controls (Fig. 8a, b). We then performed SOFG staining to detect the area and morphology of the fracture site. Relative to that of the vehicle-treated mice, the size and density of newly formed woven bone were significantly increased in GCA-NAb-treated mice (Fig. 8c, d). Furthermore, compared to the control mice, GCA-NAb-treated mice showed a decreased number of Arg2-positive cells in the callus region (Fig. 8e, f). We found that the numbers of p21^+^Ctsk^+^ periosteal stem cells and γH2AX^+^Ctsk^+^ periosteal stem cells were decreased in GCA-NAb-treated mice compared with vehicle-treated mice (Fig. 8g–i). Furthermore, GCA-NAb-treated mice showed an increased number of Ocn-positive osteoblasts in the callus (Fig. 8j, k). Collectively, these data show that the local application of GCA-NAb promotes bone-forming capacity and restores bone regeneration in aged mice.

## Discussion

**Fig. 9:**
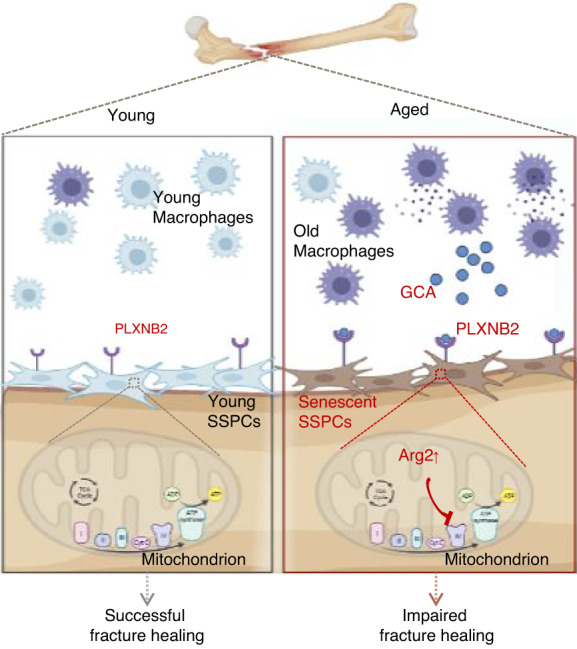
Graphical abstract of impaired fracture healing in aged mice. Schematic model for GCA-PLXNB2 transcription signaling in impaired fracture healing in aged mice

In this study, we revealed that the dysfunction and senescence of SSPCs induced by macrophage-derived GCA lead to impaired fracture healing in aged mice. Local treatment with rGCA in young mice delays fracture repair, and depletion of *Gca* in macrophages partially rejuvenates fracture repair in aged mice. We showed that GCA binds to the PLXNB2 receptor and activates Arg2-mediated mitochondrial dysfunction, resulting in SSPC senescence and defective osteochondrogenic capacity (Fig. 9). In addition, we show that the application of GCA-NAb improves bone regeneration in aged mice.

Our previous studies have found that GCA suppresses bone turnover and is mainly produced by proinflammatory and senescent subtypes of neutrophils and macrophages.^13^ In the current study, we investigated the role of GCA in fracture repair during aging. We demonstrate that deletion of the *Gca* gene in monocyte/macrophage lineage cells (*Gca-Lyz2-CKO*) mitigates SSPC senescence in the calluses of aged mice. The number of senescent cells at the fracture sites is significantly increased compared with that at nonfractured sites, indicating that acute injury may also induce cell senescence.^14^ Considering that GCA^+^ immune cells infiltrate the callus in response to fracture and that more GCA^+^ immune cells in the callus are observed during aging, we speculate that macrophages are activated in acute injury and secrete abundant GCA during aging. In addition, GCA^+^ immune cells might also migrate to the fracture site from bone marrow in aged mice. Although GCA secreted by senescent macrophages shows a negative impact on fracture healing, we recognize that GCA^+^ macrophages may have multiple effects on bone regeneration since ablation of macrophages also impairs fracture healing.^52,53^ In this study, we focused on the exact role of GCA in SSPC senescence and fracture repair, but gaining further insight into the role of GCA^+^ immune cells in age-related impaired bone regeneration will deepen the understanding of senescent immune cells in tissue repair.

PLXNB2 belongs to the plexin family, which has been reported to modulate neurogenesis, angiogenesis, immune response, tumorigenesis, and tissue regeneration.^54^ Our previous study illustrated that PLXNB2 is a functional receptor of GCA in BMSCs and plays a critical role in the osteogenic differentiation of BMSCs. PLXNB2 is widely expressed in a variety of cells and is implicated in regulating cell proliferation and migration.^55^ Additionally, plexin-B2 has been reported to protect vascular endothelial cells in the metaphysis from senescence.^11^ In this study, we found that the expression of *Plxnb2* was highest among the members of the plexin family in callus SSPCs. However, the effects of *Plxnb2* in bone regeneration have not yet been reported. Here, we show for the first time that depletion of *Plxnb2* leads to SSPC senescence, dysfunction of osteogenic and chondrogenic differentiation and impaired fracture healing. It is possible that other members of the plexin family, such as PLXNA1, with high expression in callus SSPCs also exert important effects on bone regeneration, which is worthy of future study.

In this study, we selected Ctsk as a marker of SSPCs to investigate the role of callus SSPCs in vivo, which is consistent with a previous study showing that periosteal SSPCs express Ctsk.^31,56^ Ctsk was initially identified in mature osteoclasts. To exclude the possible effects of *Plxnb2* deletion in osteoclasts on fracture repair, we used another mouse model with depletion of *Plxnb2* in Prrx1^+^ SSPCs to confirm the critical effects of *Plxnb2* in SSPCs. Both *Plxnb2-Ctsk-CKO* mice and *Plxnb2-Prrx1-CKO* mice showed impaired bone regeneration ability, which indicated the vital roles of PLXNB2 in maintaining the number and function of SSPCs during fracture healing. In addition to callus SSPCs inducing periosteal bone formation, endochondral bone formation, intramembranous bone formation and bone remodeling also contribute to fracture repair.^57^ Whether plexin-B2 is also expressed in other cell types in bone, such as endothelial cells, chondrocytes, osteoblasts, and osteocytes, thus playing a significant role in fracture repair, is an interesting topic for further investigation.

In summary, our findings highlight the critical role of the GCA-PLXNB2 axis in the crosstalk of macrophages and SSPCs in bone regeneration during aging. Whether age-related GCA accumulation affects additional biological processes involved in tissue repair requires further study. Nevertheless, our study supports the concept that senescent macrophages release GCA to induce SSPC secondary senescence and delay bone regeneration during aging, and targeting GCA may be a good strategy for promoting fracture repair in elderly individuals.

## Materials and methods

### Mice

The *Gca*-floxed mice and *Plxnb2*-floxed mice were constructed utilizing CRISPR/Cas9 technology at BIORAY LABORATORIES (China). *Lyz2-Cre* mice, *Ctsk-Cre* mice, *Prrx1-Cre* mice and *YFP* reporter mice were purchased from the Jackson Laboratory. All mice were kept on a C57BL/6 J background and maintained in a standard, specific pathogen-free facility of the Laboratory Animal Research Center of Central South University at a controlled temperature (22 °C–24 °C) with a 12 h dark/light cycle (07:00 to 19:00 light on), with standard food (Hunan SJA Laboratory Animal Company. China) and water provided ad libitum and environmental enrichments. Age- and sex-matched littermates were used as control mice. We crossed *Gca*-floxed mice with *Lyz2-Cre* mice to generate *Gca-Lyz2-CKO* mice and *Gca*^*flox/flox*^ mice. *Ctsk-Cre* mice or *Prrx1-Cre* mice were crossed with *Plxnb2*-floxed mice. The offspring were intercrossed to generate *Plxnb2-Ctsk-CKO* mice and *Plxnb2*-floxed mice or *Plxnb2-Prrx1-CKO* mice and *Plxnb2*-floxed mice. The genotypes of the mice were determined by PCR analyses of genomic DNA extracted from mouse tail snips.

### Bicortical femoral fracture

Mice were anesthetized using pentobarbital. The patella was dislocated laterally to expose the femoral condyles, and a 25-gauge regular bevel needle (BD BioSciences) was inserted to stabilize the femur. A transverse fracture was created in the mid-diaphysis using scissors. Muscles were reapproximated, and the skin was closed using a 6/0 nylon suture.

### Hydrogel placement

We purchased GelMA hydrogel from Engineering For Life Company. rGCA or PBS was added to the hydrogel solution at a concentration of 750 nmol/L. Solutions were exposed to ultraviolet light for 30 s in volumes of 10 μL each to obtain factor-loaded hydrogels.

### Microcomputed tomography

The fractured femora from mice were dissected and fixed for 24 h with 4% paraformaldehyde and then scanned and analyzed with high-resolution μCT (Skyscan 1172, Bruker MicroCT, Kontich, Belgium).^58^ The callus index (CI) of fractured bone was calculated as the maximum diameter of the callus divided by the diameter of the adjacent diaphysis.

### Histochemistry and immunofluorescence staining

Fractured bone was harvested from mice after euthanasia, fixed in 10% formalin for 12 h, and decalcified in 10% EDTA for 21 days. For histochemistry staining, the fractured bones were embedded in paraffin. Five-millimeter-thick longitudinally oriented bone sections were stained with Safranin O-Fast green (SOFG) to quantify the cartilage area and woven bone area. For immunofluorescence staining, we prepared cryo-embedding medium composed of 2% polyvinylpyrrolidone (PVP), 20% sucrose and 8% gelatin in 1× PBS. The fractured bones were embedded with cryo-embedding medium, and 7 µm cryosections were obtained. Bone sections were processed for antigen retrieval by digestion with 0.05% trypsin at 37 °C for 15 min and then incubated with primary antibodies against Ctsk (ab19027, 1:200, Abcam), p21 (sc-6246, 1:100, Santa Cruz), γ-H2AX (sc-517348, 1:100, Santa Cruz), GFP (ab6673, 1:200, Abcam), Arg2 (sc-393496, 1:100, Santa Cruz), GCA (PA5–77127, 1:200, Invitrogen), F4/80 (ab6640, 1:400, Abcam) and Ocn (M137, 1:500, Takara) overnight at 4 °C, followed by incubation with FITC- or Cy3-conjugated secondary antibodies (Jackson ImmunoResearch, 1:200). Nuclei were counterstained with DAPI (Sigma).

### SSPC isolation and culture

Femurs were dissected, cleaned of soft tissue and cut into pieces using microscissors. Then, the tissue was digested in 2.2 mg/mL collagenase II buffer at 37 °C for 60 min. The cell suspension was passed through a 70 μm nylon filter, washed in growth medium (α-MEM containing 10% FBS and 1% penicillin/streptomycin) and pelleted at 1 000 r/min. Hematopoietic lineage cells were depleted via ACK lysis for 5 min. The cells were washed again in growth medium (α-MEM containing 10% FBS and 1% penicillin/streptomycin) and pelleted at 1 000 r/min. The cell pellet was resuspended in growth medium (α-MEM containing 10% FBS and 1% penicillin/streptomycin) and plated in a 10 cm dish. All cells were grown at 37 °C in a 5% CO_2_ humid atmosphere.

### Osteogenic differentiation assay

For osteoblast differentiation, SSPCs treated with 100 nmol/L recombinant human grancalcin protein (rGCA, NBP1-50967, Novus Biologicals Inc) or transfected with siRNA-*Plxnb2* (RiboBio, China) or control SSPCs were cultured in 6/12-well plates with α-MEM containing 10% fetal bovine serum, 0.1 mmol/L dexamethasone, 10 mmol/L β-glycerol phosphate, and 50 mmol/L ascorbate-2-phosphate for 21 days. Then, the cells were stained with 2% Alizarin Red S (Sigma‒Aldrich) at pH 4.2 to evaluate cell matrix mineralization.

### Chondrogenic differentiation assay

For chondrogenic differentiation, SSPCs treated with PBS or 100 nmol/L recombinant human grancalcin protein (rGCA, NBP1-50967, Novus Biologicals Inc) or transfected with siRNA-*Plxnb2* (RiboBio, China) or control SSPCs were cultured in 6/12-well plates with DMEM containing 10% fetal bovine serum, 0.1 μmol/L dexamethasone, 50 μg/mL L-ascorbic acid and 50 mg/mL ITS.

### Western blotting analysis

Total cell lysates were separated by SDS‒PAGE and blotted on polyvinylidene difluoride membranes (Millipore). The membranes were incubated with specific antibodies against PLXNB2 (PA5-47880, 1:1 000, Invitrogen), p16 (ab211542, 1:1 000, Sigma), p21 (ZRB1141, 1:1 000, Sigma), Arg2 (A6355, 1:1 000, ABclonal), GCA (PA5-77127, 1:800, Invitrogen), and GAPDH (5174, 1:2 000, Cell Signaling Technology) and then reprobed with appropriate horseradish peroxidase-conjugated secondary antibodies. Blots were visualized by enhanced chemiluminescence (ECL Kit; Amersham Biosciences).

### qRT‒PCR analysis

qRT‒PCR analysis was performed using a Roche Molecular Light Cycler (Roche) as previously described.^59^ Total RNA from cells was isolated using TRIzol reagent (Invitrogen). Reverse transcription was performed using 1 mg total RNA and Super-Script II (Invitrogen). Amplification reactions were set up in 25 mL reaction volumes containing SYBR Green PCR Master Mix (PE Applied Biosystems), a 1 mL volume of cDNA, and amplification primers.

### Mitochondrial membrane potential assay

SSPCs were incubated with PBS or 100 nmol/L recombinant human grancalcin protein (rGCA, NBP1-50967, Novus Biologicals Inc.) for 48 h. Cells were stained using the Mitochondrial Membrane Potential Assay Kit with JC-1 (Beyotime).

### RNA sequencing

The RNA-sequencing assay was performed by Personalbio. RNA was extracted from PBS- or rGCA-treated SSPCs and siRNA-*Plxnb2-* or siRNA-NC-transfected SSPCs after 36 h of treatment. Differential gene expression analyses were conducted using DESeq with the criteria of fold change >2 and *P-value* < 0.05. The RNA sequencing data used in this article are available on the Sequence Read Archive website with the BioProject accession number PRJNA987851.

### Statistical analysis

Data are presented as the mean ± SD. A Shapiro‒Wilk test for normality was performed on all datasets. Homogeneity was confirmed by a comparison of variances test. Parametric data were analyzed using an appropriate two-sided Student’s *t* test or Welch’s *t* test when two groups were being compared. Nonparametric data were analyzed with a Mann‒Whitney U test when two groups were being compared. Differences were considered significant at *P* < 0.05. The sample size for in vivo and in vitro experiments was based on previous experience. All the samples were randomly assigned, and none of the experiments in the study were performed in a blinded fashion. For both in vitro and in vivo experiments, no initial exclusion criteria were used, and no animals or replicates were excluded from the study.

### Supplementary information

Supplementary figures_1

Supplementary figures_2

Supplementary figures_3

Supplementary figures_4

Supplementary figures_5

Supplementary figures_6

Supplementary figures_7

Supplementary figures
