# Supplementary figures and images for: Age-related secretion of grancalcin by macrophages induces skeletal stem/progenitor cell senescence during fracture healing
Source: Bone Res. 2024 Jan 25;12:6. doi: 10.1038/s41413-023-00309-1 (PMC10808101; doi:10.1038/s41413-023-00309-1)

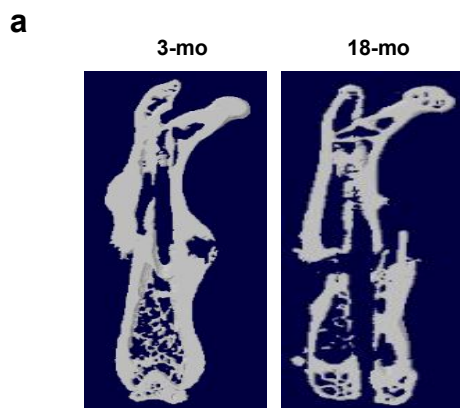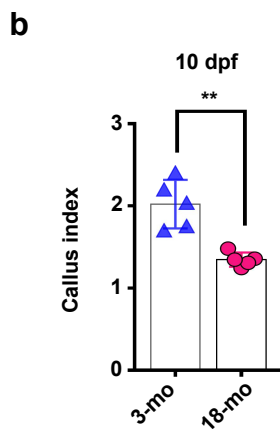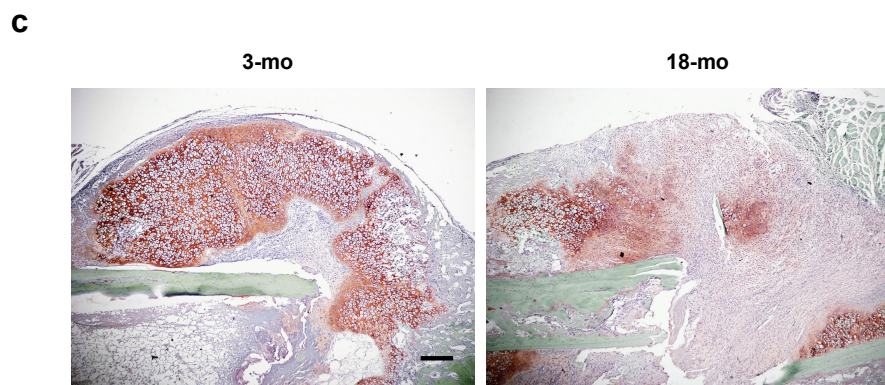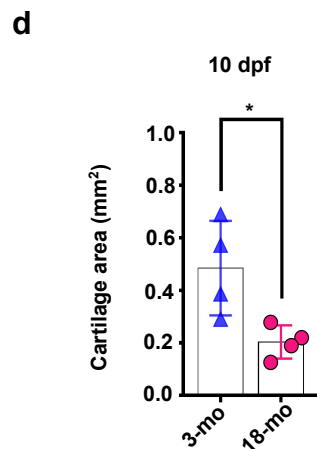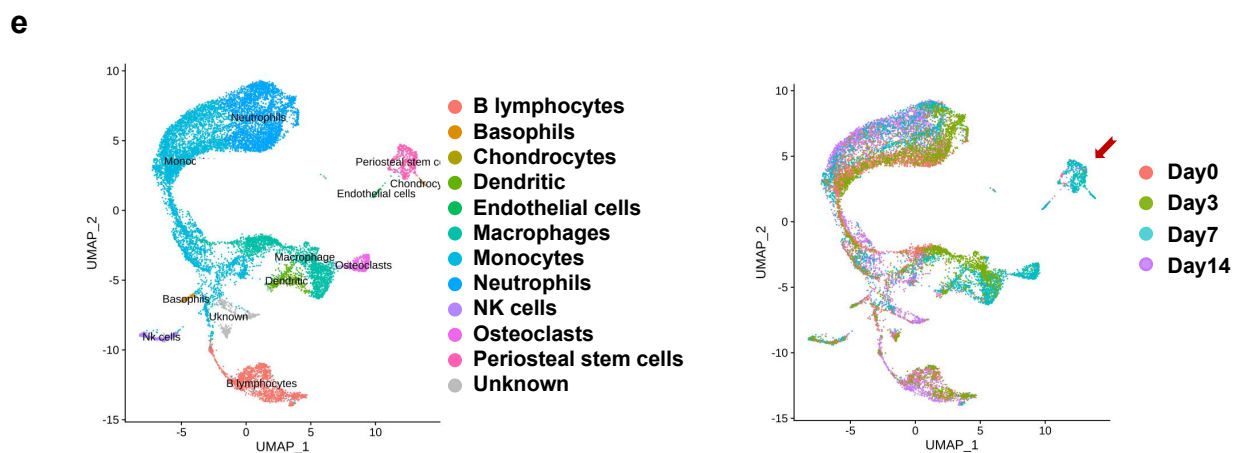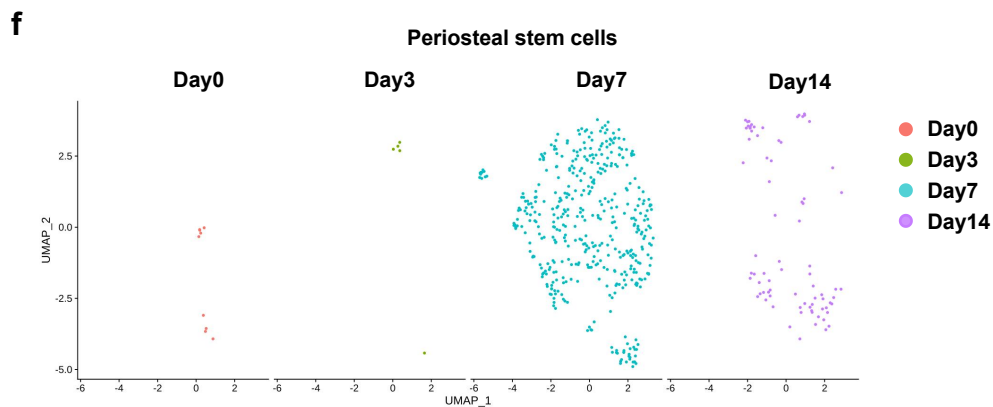

Supplement: Supplementary file 1 — Supplementary figures_1 [file 41413_2023_309_MOESM1_ESM.pdf]

**a**

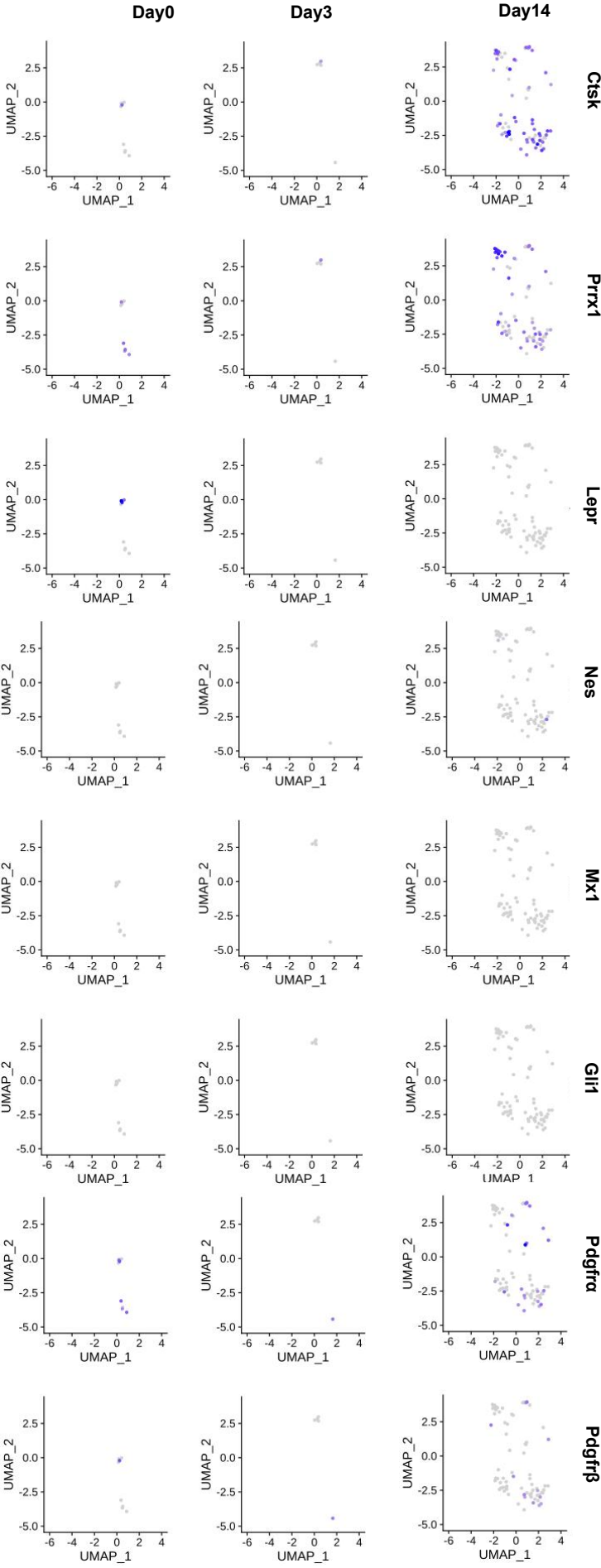

**b**

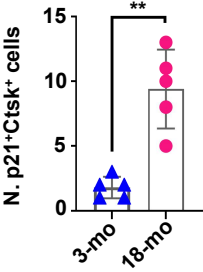

**c**

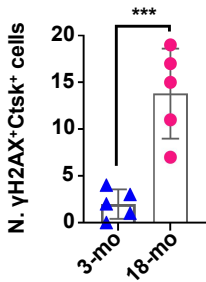

Supplement: Supplementary file 2 — Supplementary figures_2 [file 41413_2023_309_MOESM2_ESM.pdf]

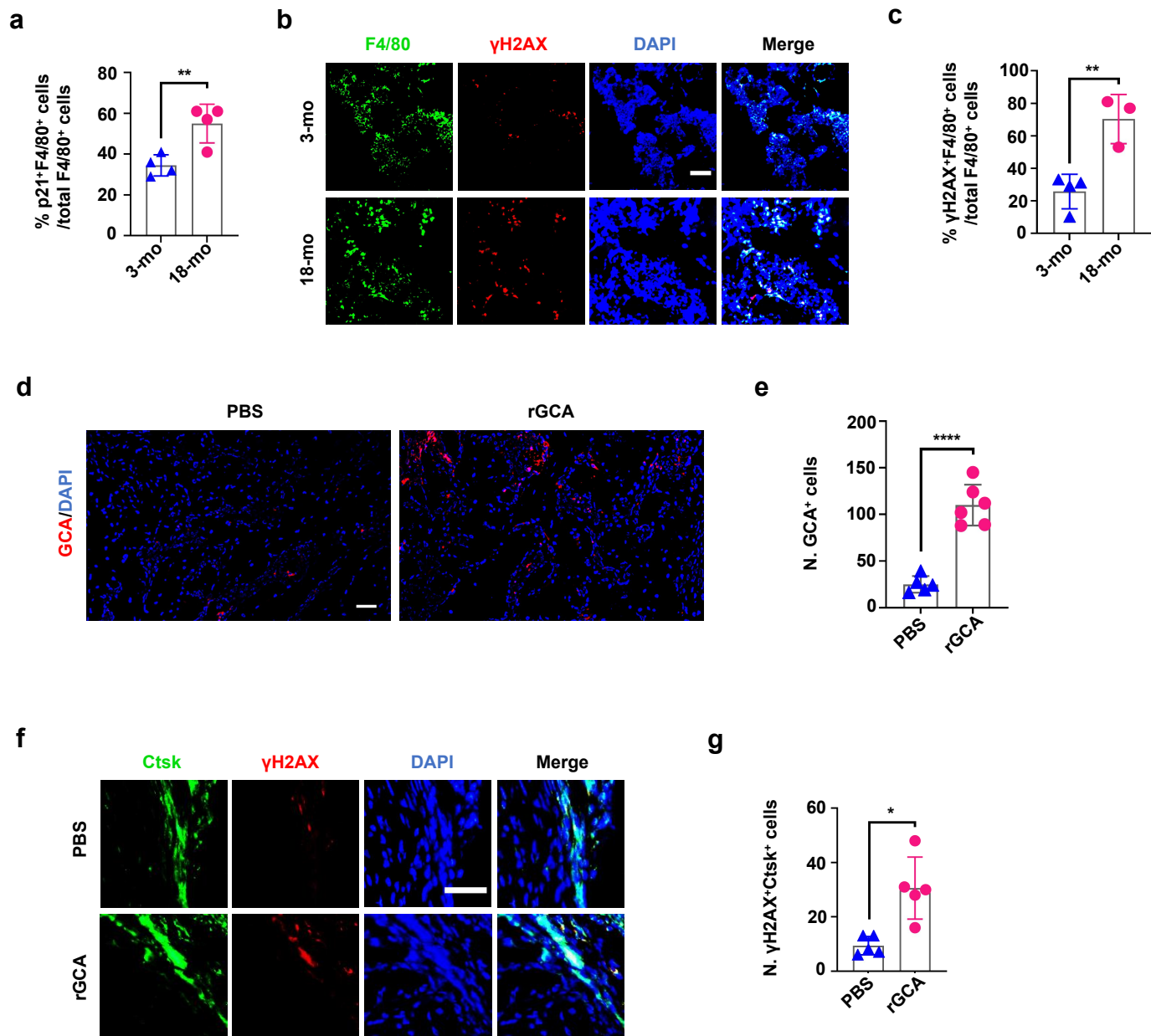

Supplement: Supplementary file 3 — Supplementary figures_3 [file 41413_2023_309_MOESM3_ESM.pdf]

**a**

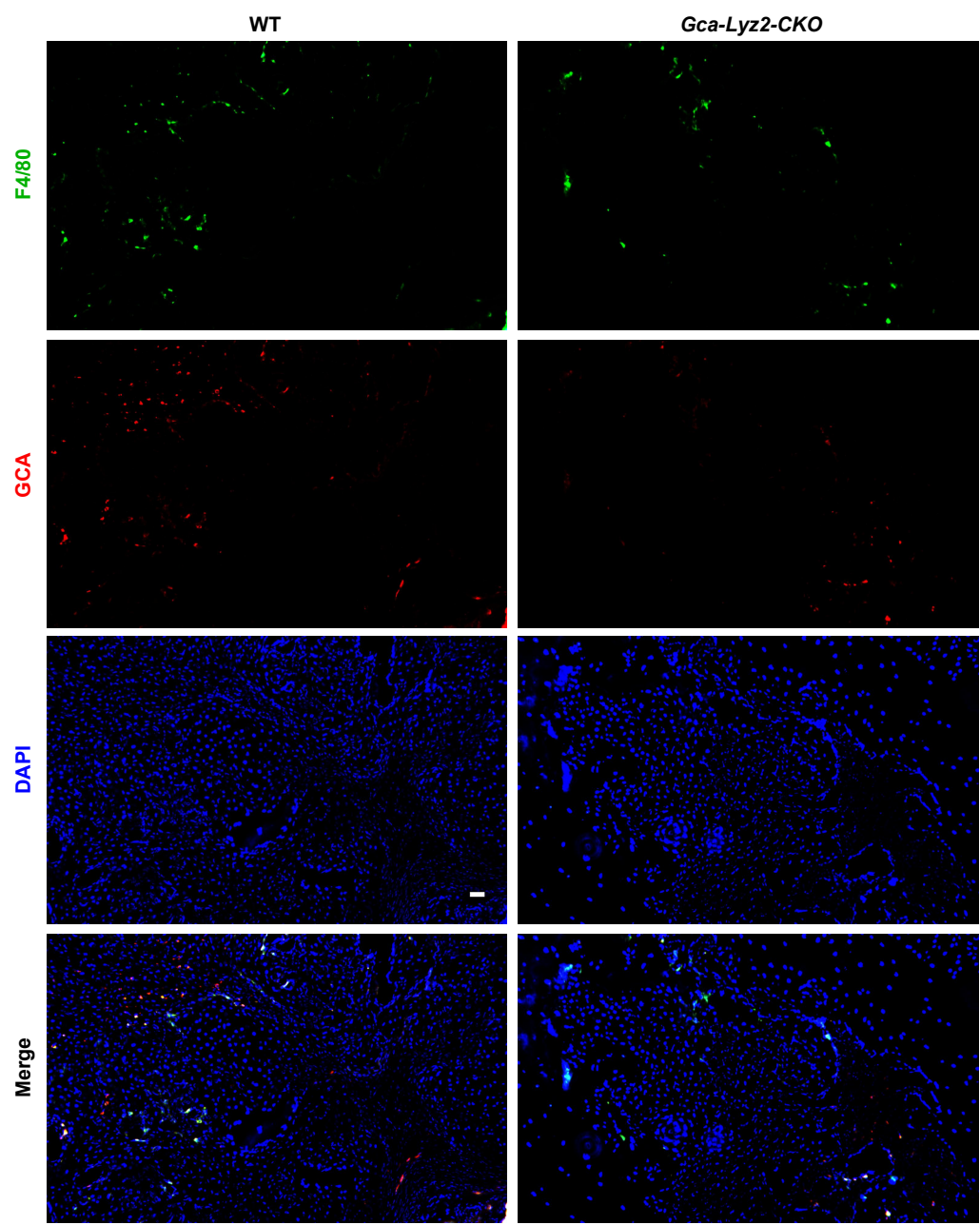

**b**

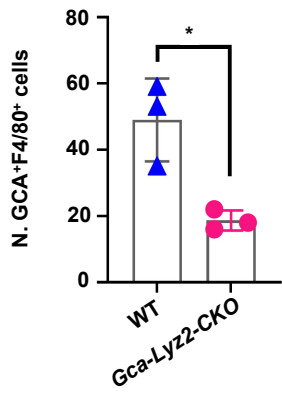

Supplement: Supplementary file 4 — Supplementary figures_4 [file 41413_2023_309_MOESM4_ESM.pdf]

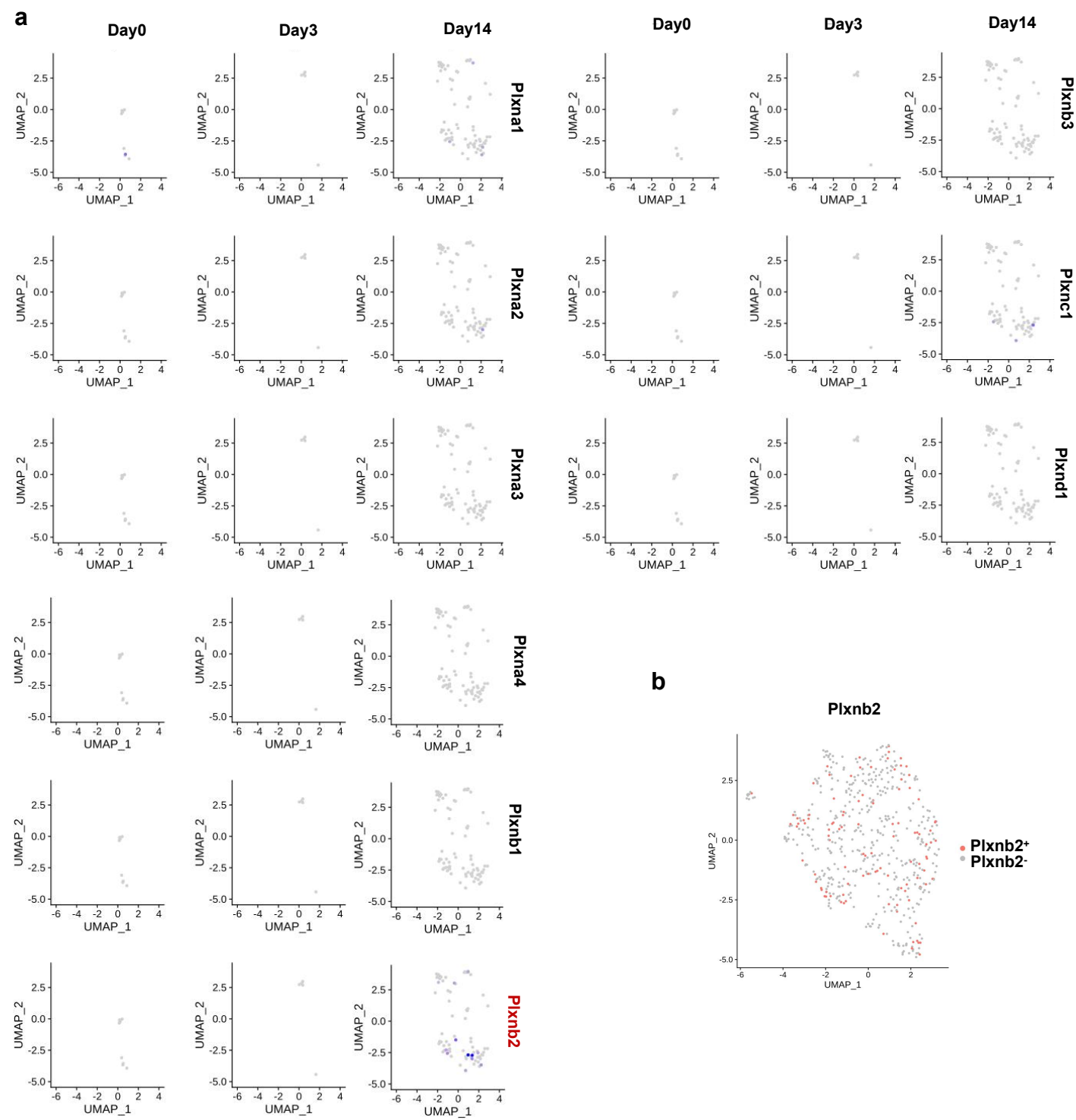

**b**

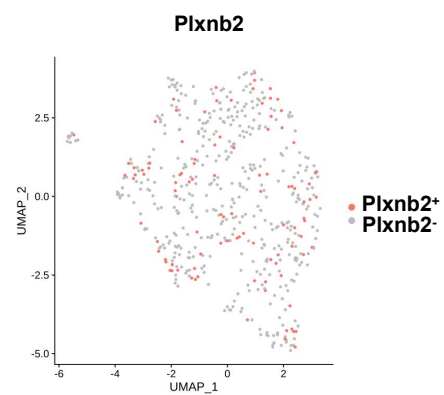

**c**

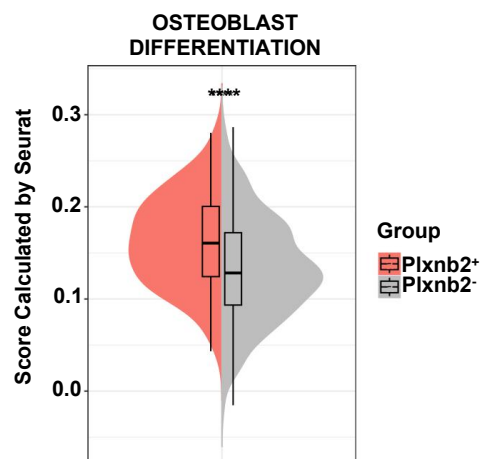

**d**

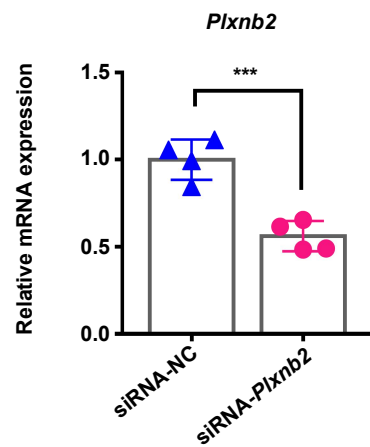

Supplement: Supplementary file 5 — Supplementary figures_5 [file 41413_2023_309_MOESM5_ESM.pdf]

**a**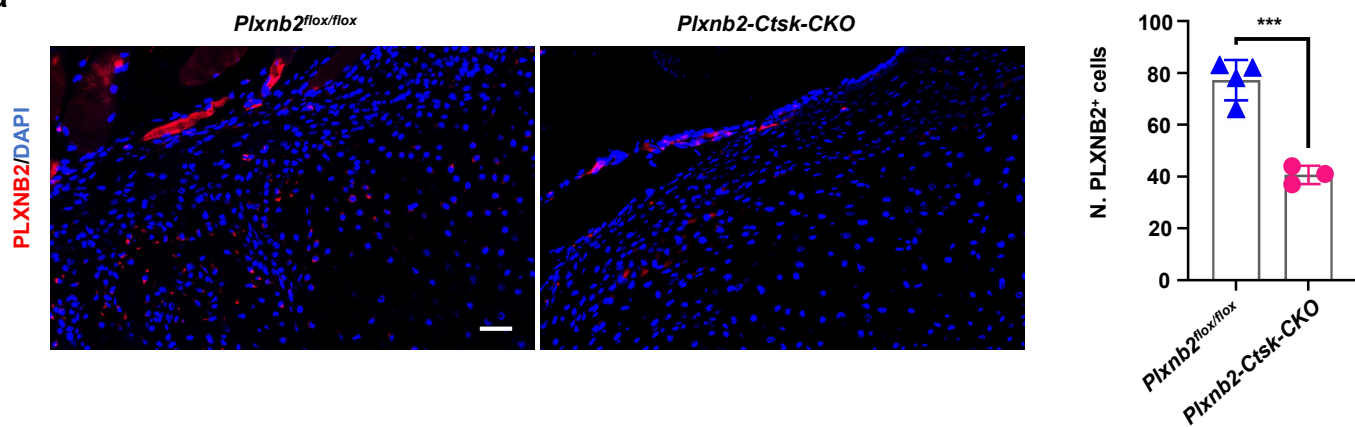**b**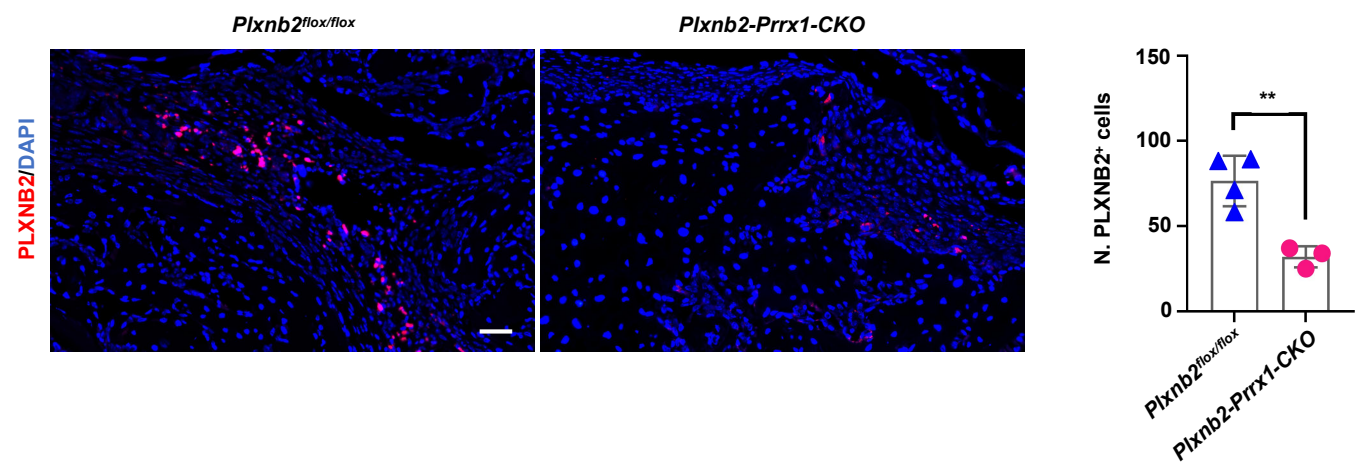**c**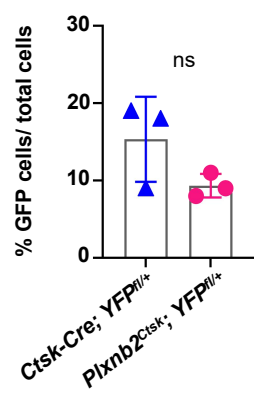

Supplement: Supplementary file 6 — Supplementary figures_6 [file 41413_2023_309_MOESM6_ESM.pdf]

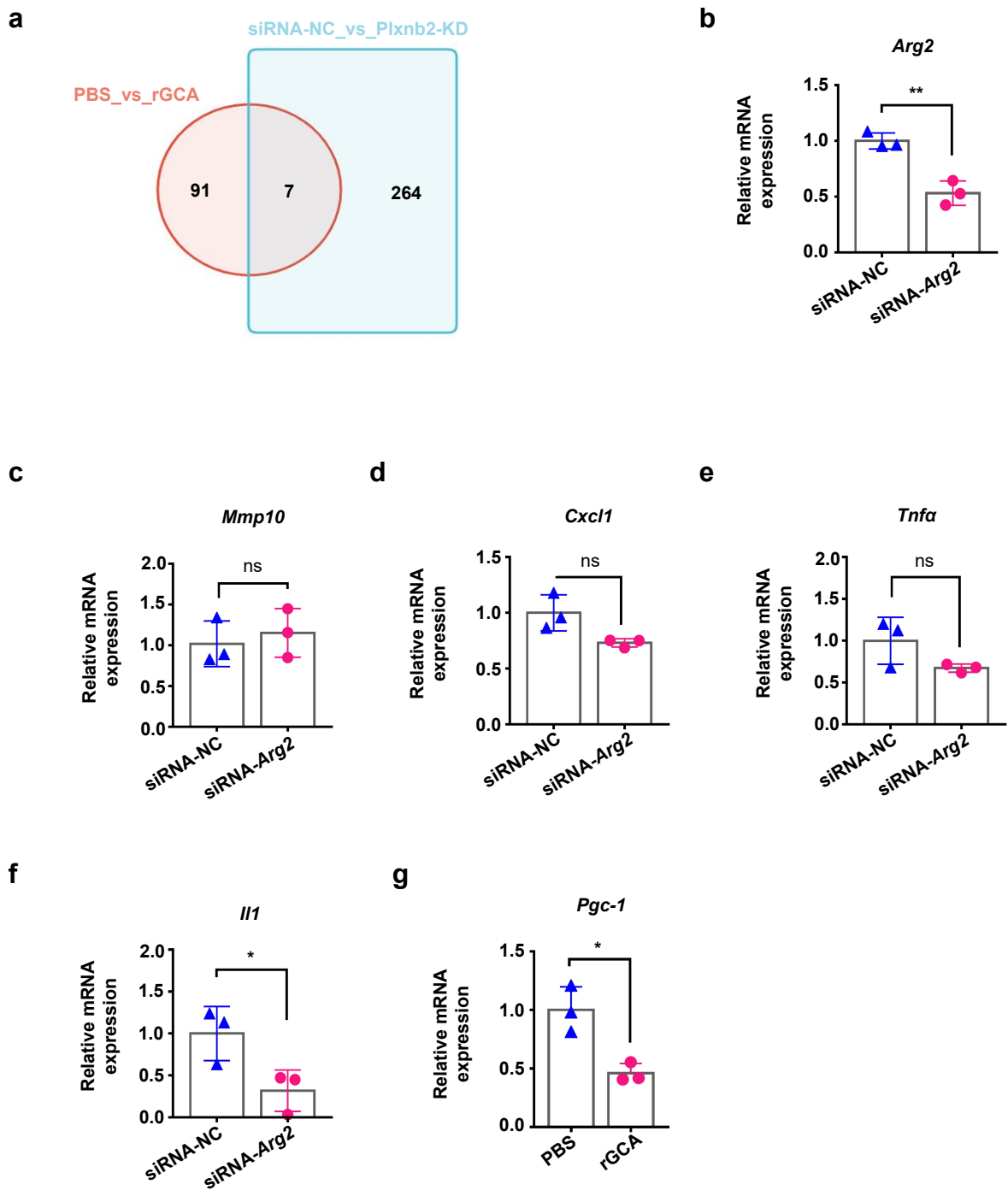

Supplement: Supplementary file 7 — Supplementary figures_7 [file 41413_2023_309_MOESM7_ESM.pdf]
