# Supplementary material for: Age-related secretion of grancalcin by macrophages induces skeletal stem/progenitor cell senescence during fracture healing
Source: Bone Res. 2024 Jan 25;12:6. doi: 10.1038/s41413-023-00309-1 (PMC10808101; doi:10.1038/s41413-023-00309-1)
Supplement: Supplementary file 8 — Supplementary figures [file 41413_2023_309_MOESM8_ESM.docx]

**Figure S1 (Related to Figure 1) Clusters in fracture calluses from young and aged mice. a** Representative micro-CT images of fractured femurs in 3-month-old and 18-month-old mice at 10 dpf. **b** The callus index of fractured femurs from 3-month-old and 18-month-old mice at 10 dpf (n=5). **c, d** Safranin O staining showing cartilage callus formation in fractured femurs from 3-month-old and 18-month-old mice at 10 dpf (n = 4). Scale bar indicates 100 μm. **e** UMAP plot showing 12 distinct clusters of cells identified and color-coded from mouse fracture models. **f** UMAP plot showing periosteal stem cells identified and color-coded from mouse fracture models at different stages (control group and on Days 3, 7, and 14). Data are presented as the means ± SDs. Unpaired t test, *p < 0.05 and **p < 0.01.

**Figure S2 (Related to Figure 1) SSPC profile in fracture calluses from young and aged mice. a** Feature plots showing the expression distribution of marker genes of SSPC subsets on Days 0, 3, and 14. Expression levels for each cell are color-coded and overlaid onto the UMAP plot. **b, c** Quantification of the number of p21- and Ctsk-positive cells per mm^2^ tissue area (N. p21^+^Ctsk^+^ cells) by Welch’s t test and the number of γH2AX- and Ctsk-positive cells per mm^2^ tissue area (N. γH2AX^+^Ctsk^+^ cells) by unpaired t test are shown in (**b**) and (**c**), respectively (n = 5). Data are presented as the means ± SDs. **p < 0.01 and ***p < 0.001.

**Figure S3 (Related to Figure 2) GCA accumulated in BMMs during aging. a** Quantification of p21^+^F4/80^+^ cells (n=4). **b** Representative IF images of fracture calluses immunostained with F4/80 (green) and γH2AX (red) antibodies and counterstained with DAPI (blue). Scale bars indicate 100 µm. **c** Quantification of γH2AX^+^F4/80^+^ cells (n=3-4). **d** Representative IF images of fracture calluses immunostained with GCA (red) antibodies and counterstained with DAPI (blue). Scale bars indicate 100 µm. **e** Quantification of GCA^+^ cells (n=5-6). **f** Representative IF images of fracture calluses immunostained with Ctsk (green) and γH2AX (red) antibodies and counterstained with DAPI (blue). Scale bars indicate 100 µm. **g** Quantification of γH2AX^+^Ctsk^+^ cells (n=5). Data are presented as the means ± SDs. Unpaired t test, *p < 0.05, **p < 0.01, ***p < 0.001 and ****p < 0.0001.

**Figure S4 (Related to Figure 4) Conditional deletion efficiency in *Gca-Lyz2-CKO* mice. a** Representative IF images of fracture calluses immunostained with F4/80 (green) and GCA (red) antibodies and counterstained with DAPI (blue). Scale bars indicate 100 µm. **b** Quantification of GCA^+^F4/80^+^ cells (n=3). Data are presented as the means ± SDs. Unpaired t test, *p < 0.05.

**Figure S5 (Related to Figure 5) Function and distribution of *Plxnb2* in SSPCs. a** Feature plots showing the expression distribution of the plexin family on Days 0, 3, and 14. Expression levels for each cell are color-coded and overlaid onto the UMAP plot. **b** Feature plots showing the expression distribution of *Plxnb2*. Expression levels for each cell are color-coded and overlaid onto the UMAP plot. **c** Violin plots demonstrating that *Plxnb2*^+^ SSPCs positively correlate with gene sets for osteogenesis. **d** QPCR analysis of *Plxnb2* mRNA expression levels in siRNA-NC- and siRNA-*Plxnb2*-transfected SSPCs (n=4). Data are presented as the means ± SDs. Unpaired t test, ***p < 0.001.

**Figure S6 (Related to Figure 6) Conditional deletion efficiency of *Plxnb2*. a, b** Representative IF images of fracture calluses immunostained with PLXNB2 (red) antibodies and counterstained with DAPI (blue) (**a**) and quantification of PLXNB2^+^ cells (n=3-4) (**b**). Scale bars indicate 100 µm. Data are presented as the means ± SDs. Unpaired t test, **p < 0.01, ***p < 0.001.

**Figure S7 (Related to Figure 7) The effects of Arg2 on inducing SSPC aging. a** Venn diagram of differentially expressed genes in the rGCA and/or siRNA-*Plxnb2* groups. **b-f** QPCR analysis of *Arg2* and SASP factor mRNA expression levels in siRNA-NC- and siRNA-*Arg2*-transfected SSPCs. **g** QPCR analysis of *Pgc-1* gene expression in PBS- and rGCA-treated SSPCs (n=3). Data are presented as the means ± SDs. Unpaired t test, *p < 0.05, **p < 0.01 and ns = no significant difference.
